# Supplementary figures and images for: Characterization of a new CCCTC-binding factor binding site as a dual regulator of Epstein-Barr virus latent infection
Source: PLoS Pathog. 2023 Jan 25;19(1):e1011078. doi: 10.1371/journal.ppat.1011078 (PMC9876287; doi:10.1371/journal.ppat.1011078)

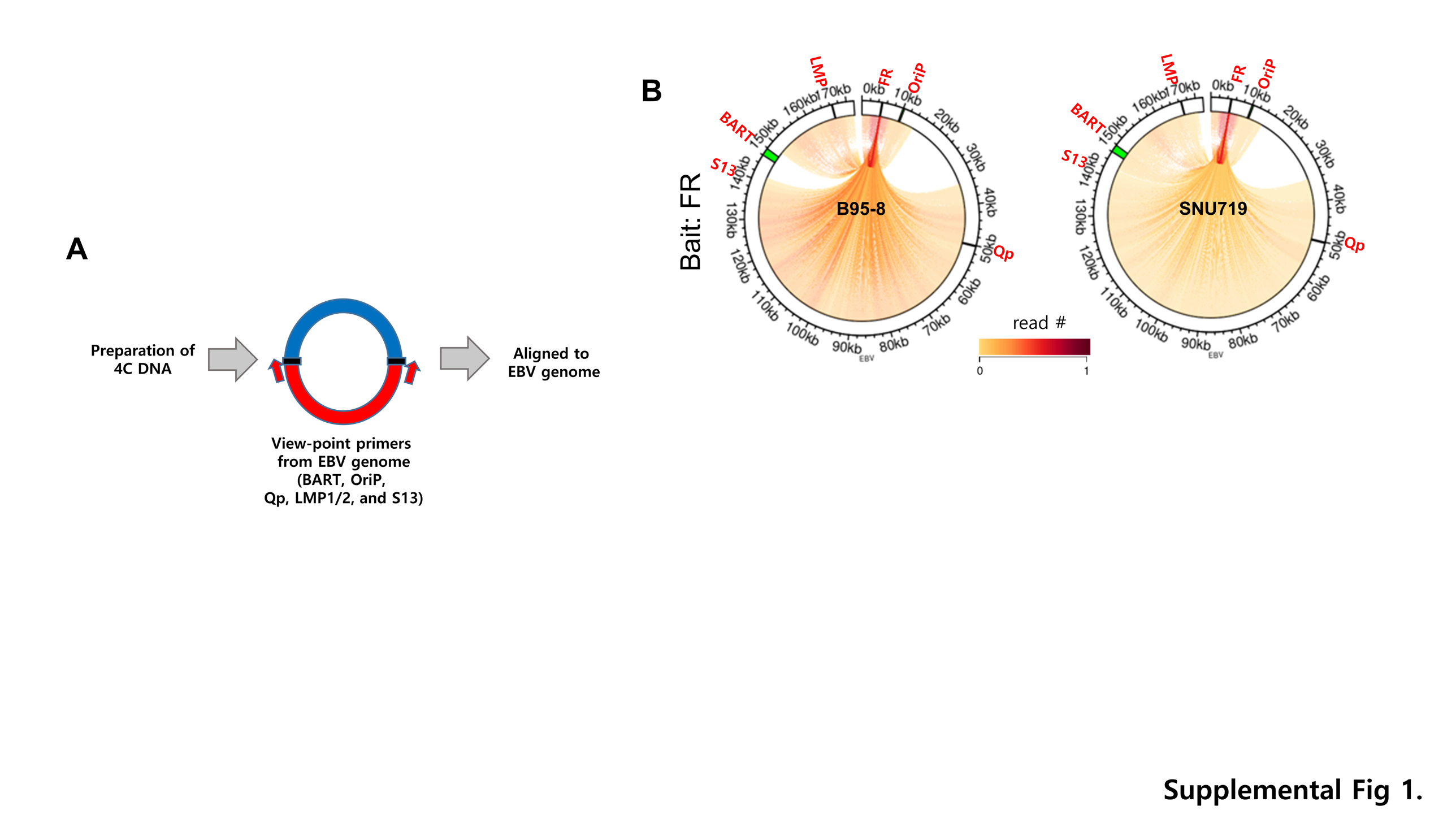

Supplement: S1 Fig — A) Four genomic loci (BART, OriP, Qp, and LMP1/2) were used as baits. The viewpoint primer sets are listed in S3 Table. B) 4C-seq assays revealed all interactions of FR regions with other loci in EBV genomes in B95-8 cells and SNU719 cells. The FR viewpoint primer sets were located at EBV 4731–4751 and EBV 4941–4961. (TIF) [file ppat.1011078.s001.TIF]

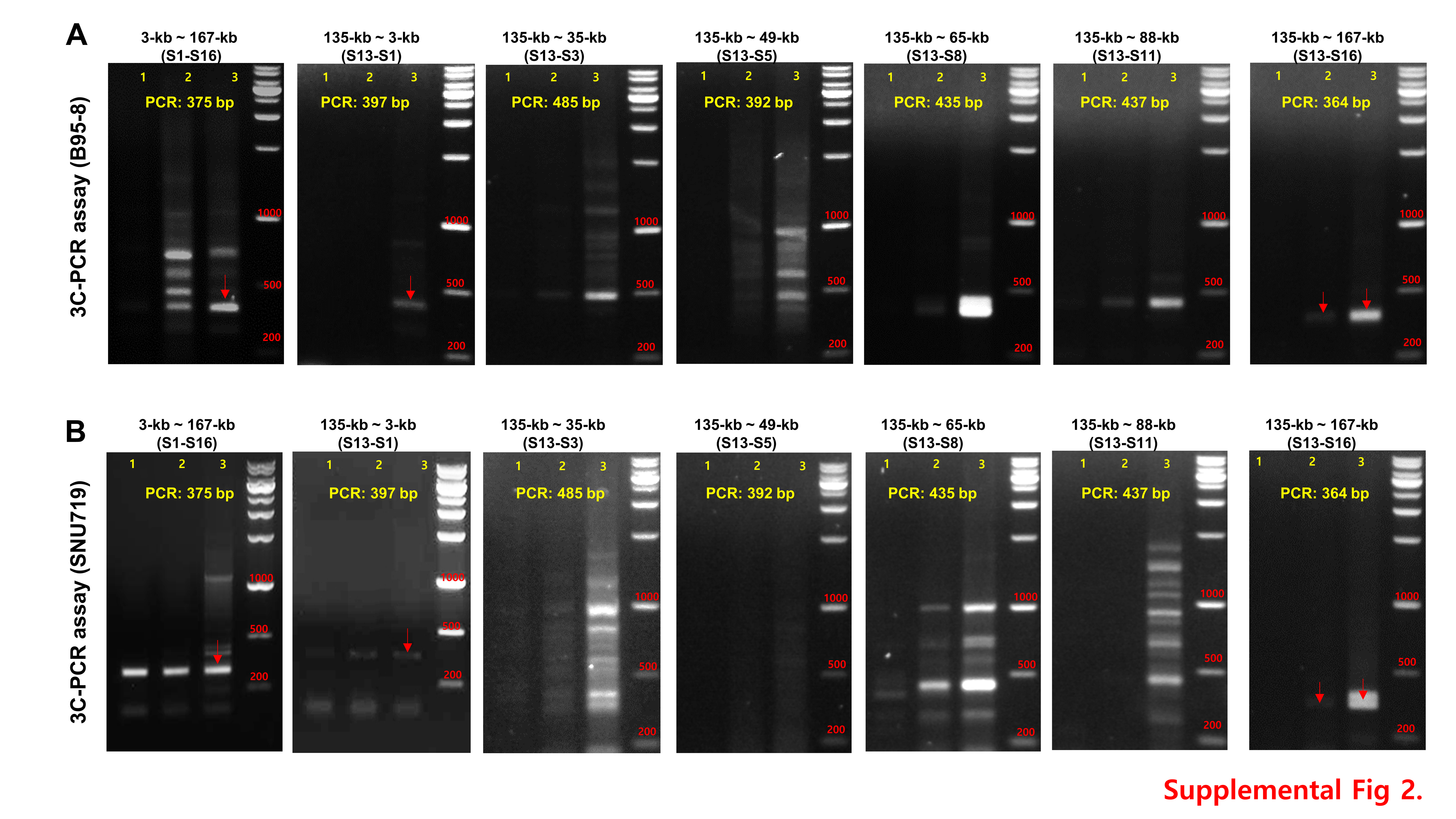

Supplement: S2 Fig — Significant genomic associations of target viewpoint regions in B95-8 and SNU719 EBV genomes were identified by 3C-PCR assays. To verify EBV genomic associations defined by 4C-seq analysis, 3C-PCR assay was conducted using B95-8 (A) and SNU719 (B) cells. Linked associations between the bait and target regions were amplified as PCR products in the 3C-PCR assay. 3C DNA products were prepared and subjected to PCR assay using 0.5 μg (label-1), 5 μg (label-2), and 50 μg (label-3) of 3C DNA products as template to determine DNA associations within EBV genomic loci. The bait region was the 135-kb locus adjacent to S13. Target region sizes were 3-kb (near S1), 35-kb (near S3), 49-kb (near S5), 88-kb (near S11), and 167-kb (near S16), respectively. The tiny arrow indicates the PCR product suggesting association between the 135-kb region and one of the target regions in the 3C-PCR assay. 3-kb & 167-kb association was tested by 3C-PCR assay with OHK649 and OHK648 primer set, 135-kb & 3-kb association with OHK728 and OHK649 primer set, 135-kb & 35-kb association with OHK728 and OHK687 primer set, 135-kb & 49-kb association with OHK728 and OHK683 primer set, 135-kb & 65-kb association with OHK728 and OHK689 primer set, 135-kb & 88-kb association with OHK728 and OHK691 primer set, and 135-kb & 167-kb association with OHK728 and OHK648 primer set. (TIF) [file ppat.1011078.s002.TIF]

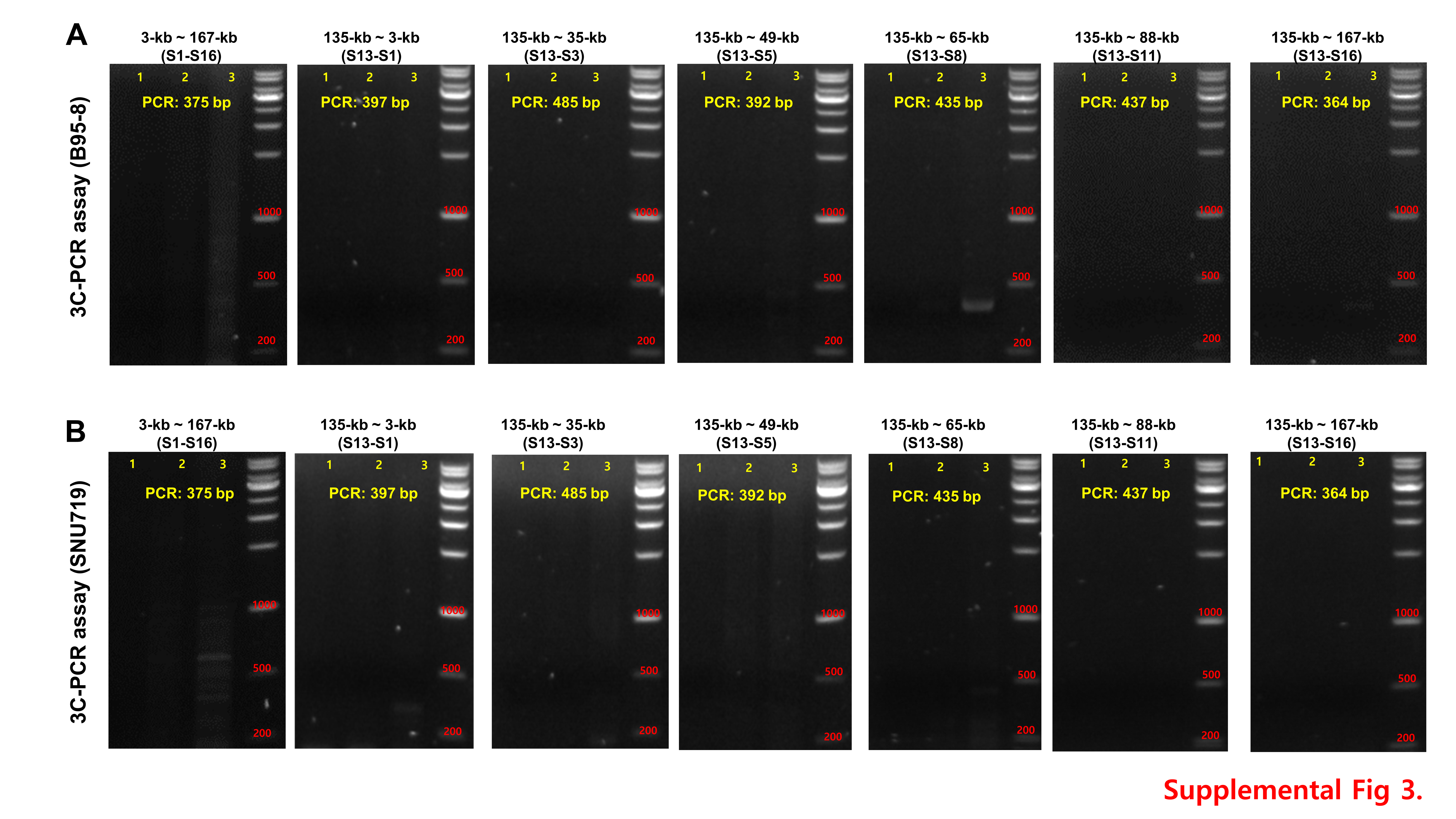

Supplement: S3 Fig — Negative experiments in 3C-PCR assay for analysis of EBV genomic associations were conducted using unligated XhoI-digested B95-8 (A) and SNU719 (B) DNA samples under the same conditions as the PCR assay with ligated XhoI-digested B95-8 and SNU719 DNA samples. Unligated XhoI-digested B95-8 and SNU719 DNA samples were not subjected to T4 DNA ligase mediated ligations. Unligated DNA samples were subjected to PCR assay using 0.5 μg (label-1), 5 μg (label-2), and 50 μg (label-3) of unligated DNA samples as template to determine false-positive amplification from primer sets in 3C-PCR assays. PCR primer sets used in analyzing ligated DNA samples were equally used exploited to negative experiments using unligated DNA samples. This PCR assay with unligated DNA samples were considered as negative control experiment to assess false positivity of 3C-PCR assay with ligated DNA samples. (TIF) [file ppat.1011078.s003.TIF]

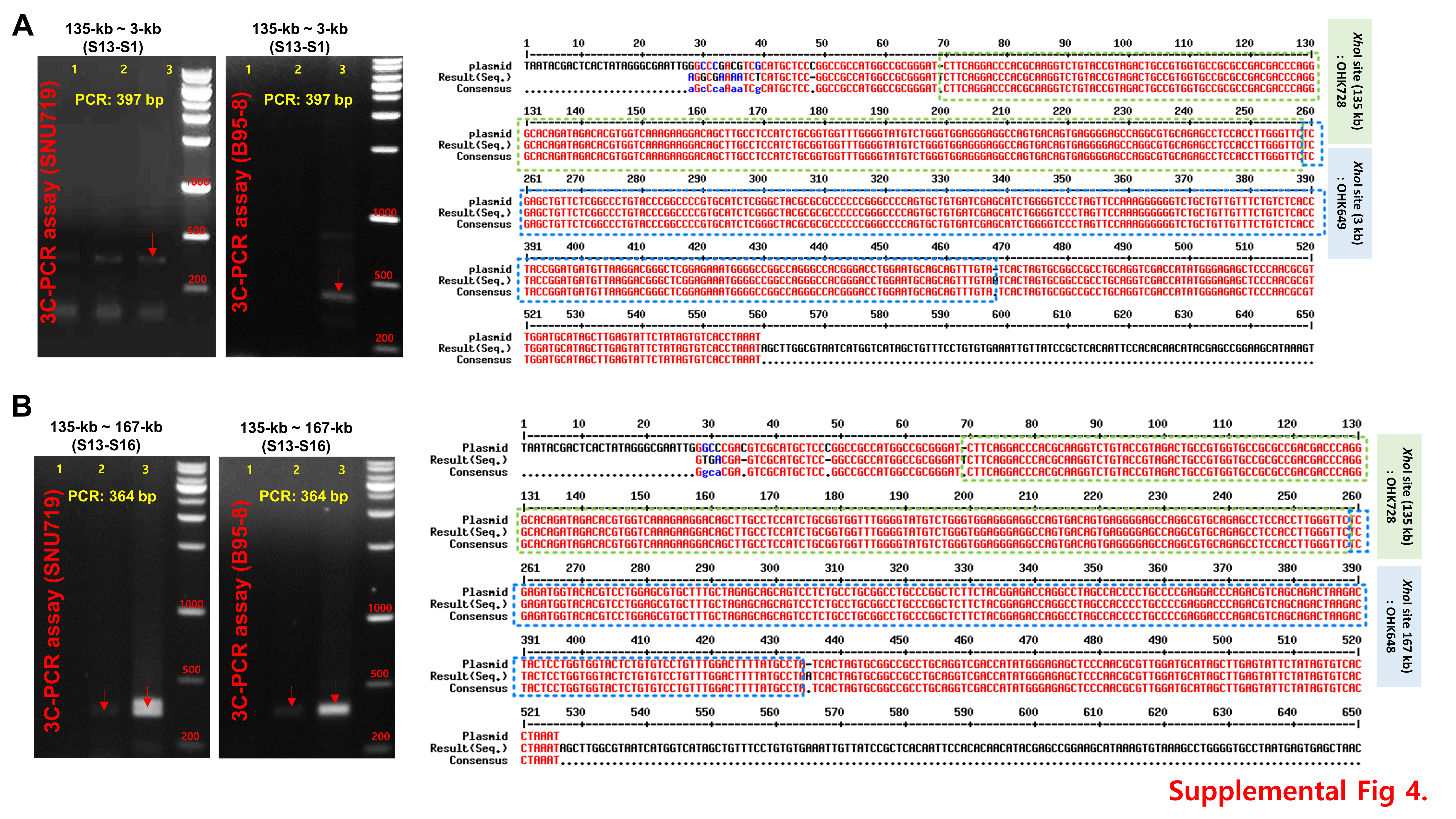

Supplement: S4 Fig — DNA fragments amplified from 3C-PCR assay were verified to link with S13 locus (EBV genome 135-kb region). A) 397-bp DNA (S13 locus) fragment indicating the linkage of 135-kb region to 3-kb region (S1 locus) was cloned to pGEM-T vector. The cloned T vector was subjected to sequence insert DNA fragments using T7 or Sp6 primer sets. B) 364-bp DNA fragment indicating the linkage of 135-kb region to 167-kb region (S16 locus) was cloned to pGEM-T vector. The cloned T vector was subjected to sequence insert DNA fragments using T7 or Sp6 primer sets. (TIF) [file ppat.1011078.s004.TIF]

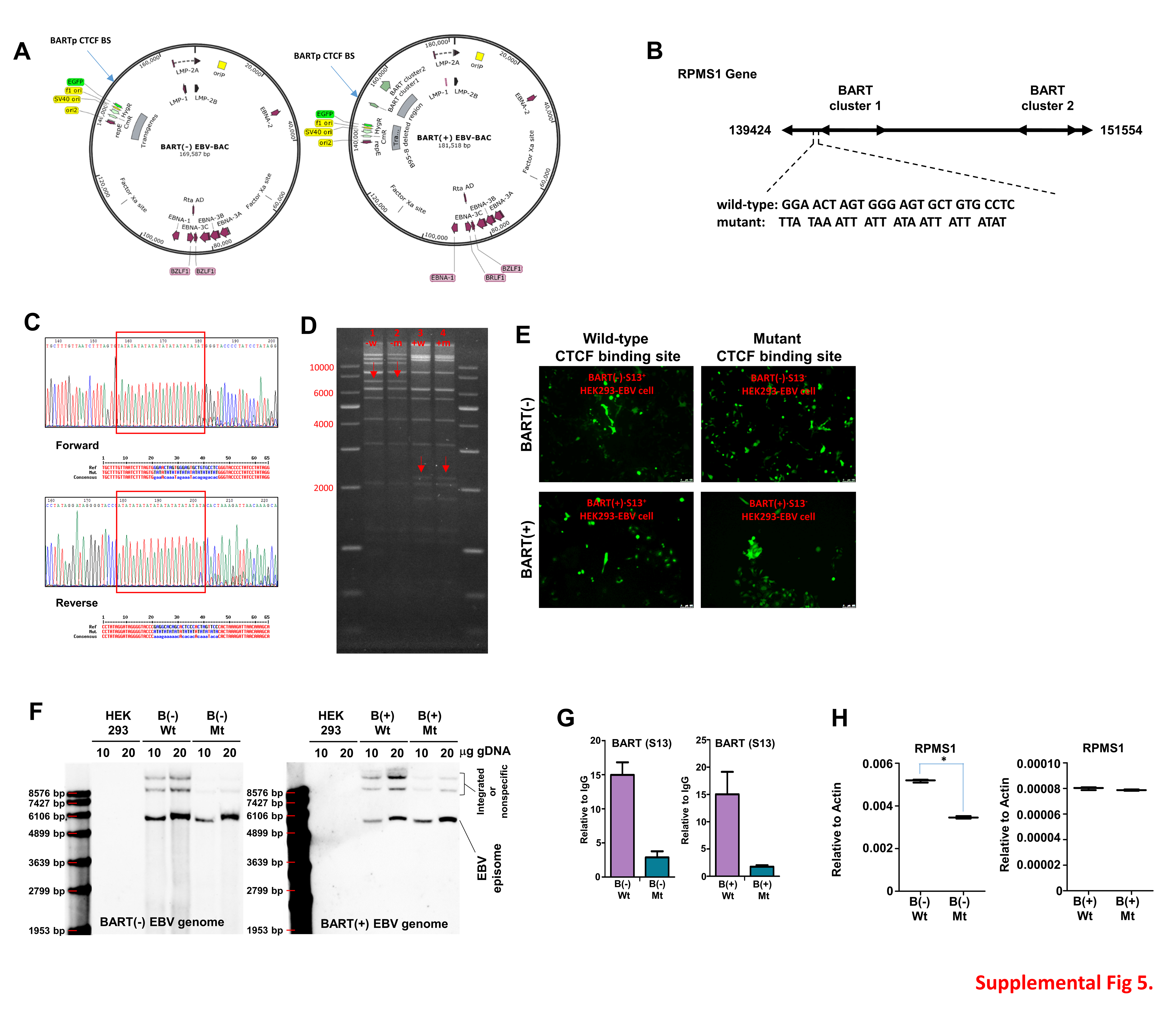

Supplement: S5 Fig — A) Schematic diagram of BART(+/-)·S13+ EBV bacmids. A CTCF-binding site on the BART miRNA promoter was identified as BARTp CTCF BS. B) Sequences introduced at the points of recombination to introduce site-directed mutations at the S13 locus (EBV 138963–138987). C) Confirmation of the site-directed mutation in the S13 locus in the Mt BART(+)·S13- EBV bacmid by Sanger sequencing. The Mt BART(-)·S13- EBV bacmid was also confirmed by Sanger sequencing. D) Gel electrophoresis to check stabilities of EBV genome in EBV bacmids following EcoRI digestion; BART(-)·S13+ EBV bacmid (lane 1, -w), Mt BART(-)·S13- EBV bacmid (lane 2, -m), BART(+)·S13+ EBV bacmid (lane 3, +w), and Mt BART(+)·S13- EBV bacmid (lane 4, +m). Bands generated by EcoRI digestion of both BART(-)·S13+ and Mt BART(-)·S13- EBV bacmids are marked on lanes 1 and 2 of the gel by the top two arrows. Other bands specific to EcoRI digestion of both BART(+)·S13+ and Mt BART(+)·S13- EBV bacmids are marked on lanes 3 and 4 of the gel by the bottom two arrows. E) Establishment of BART(+/-)·S13 (HEK293-EBV BART(-)·S13+, HEK293-EBV BART(-)·S13-, HEK293-EBV BART(+)·S13+, and HEK293-EBV BART(+)·S13-) HEK293-EBV cells. HEK293 cells were transfected with all four types of BART(+/-)·S13 (BART(-)·S13+, BART(-)·S13-, BART(+)·S13+, and BART(+)·S13-) EBV plasmids and selected using hygromycin B to establish all four types of BART(+/-)·S13 HEK293-EBV cells. GFP expression was determined 40 d after hygromycin B selection for several passages. Established BART(+/-)·S13 HEK293-EBV cells maintained GFP expression even after several passages. F) Southern blot analysis was conducted to test if BART(+/-)·S13 EBV bacmids established their episomes in HEK293 cells. Briefly, genomic DNAs from all four types of BART(+/-)·S13 HEK293-EBV cells were digested with EcoRI, purified with Phenol/chloroform/isoamyl alcohol solution treatment, and run on a gel. DNA probe was formed from EBV 78803 to 79522, and genomic DNA from HEK293 cells was used as [file ppat.1011078.s005.TIF]

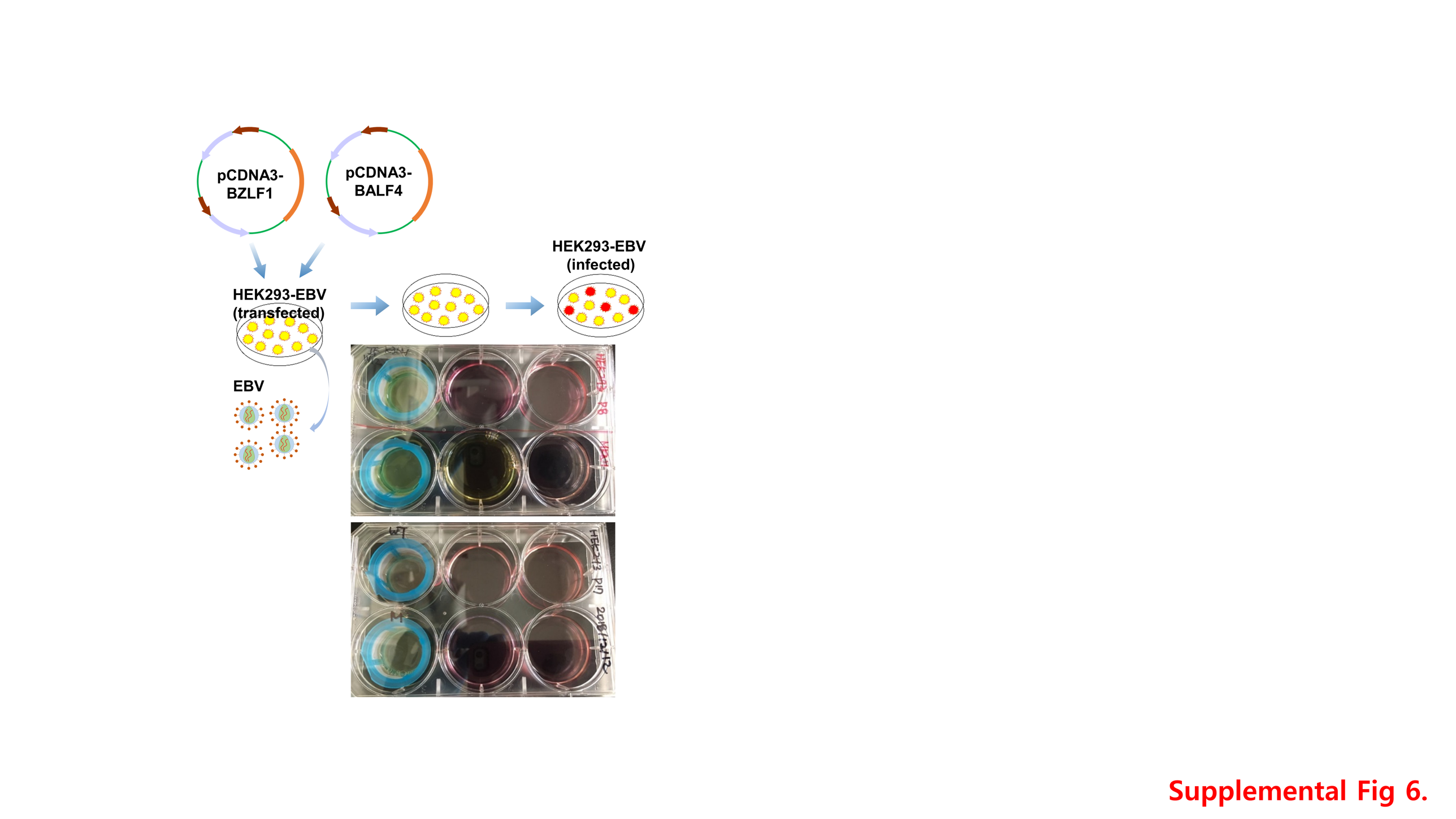

Supplement: S6 Fig — BART(+/-)·S13+ and Mt BART(+/-)·S13- HEK293-EBV cells were transfected with pCDNA3-BZLF1 and pcDNA3-BALF4. At three days post-transfection, supernatants of all four types of transfected BART(+/-)·S13 HEK293-EBV cells were harvested and added to HEK293 cells freshly cultured on a 6-well plate where a cell strainer was placed to remove cell debris. EBV in harvested supernatants were allowed to infect HEK293 cells for 24 h. After infection, EBV-encoded GFP in infected HEK293 cells was detected over a series of time points. (TIF) [file ppat.1011078.s006.TIF]

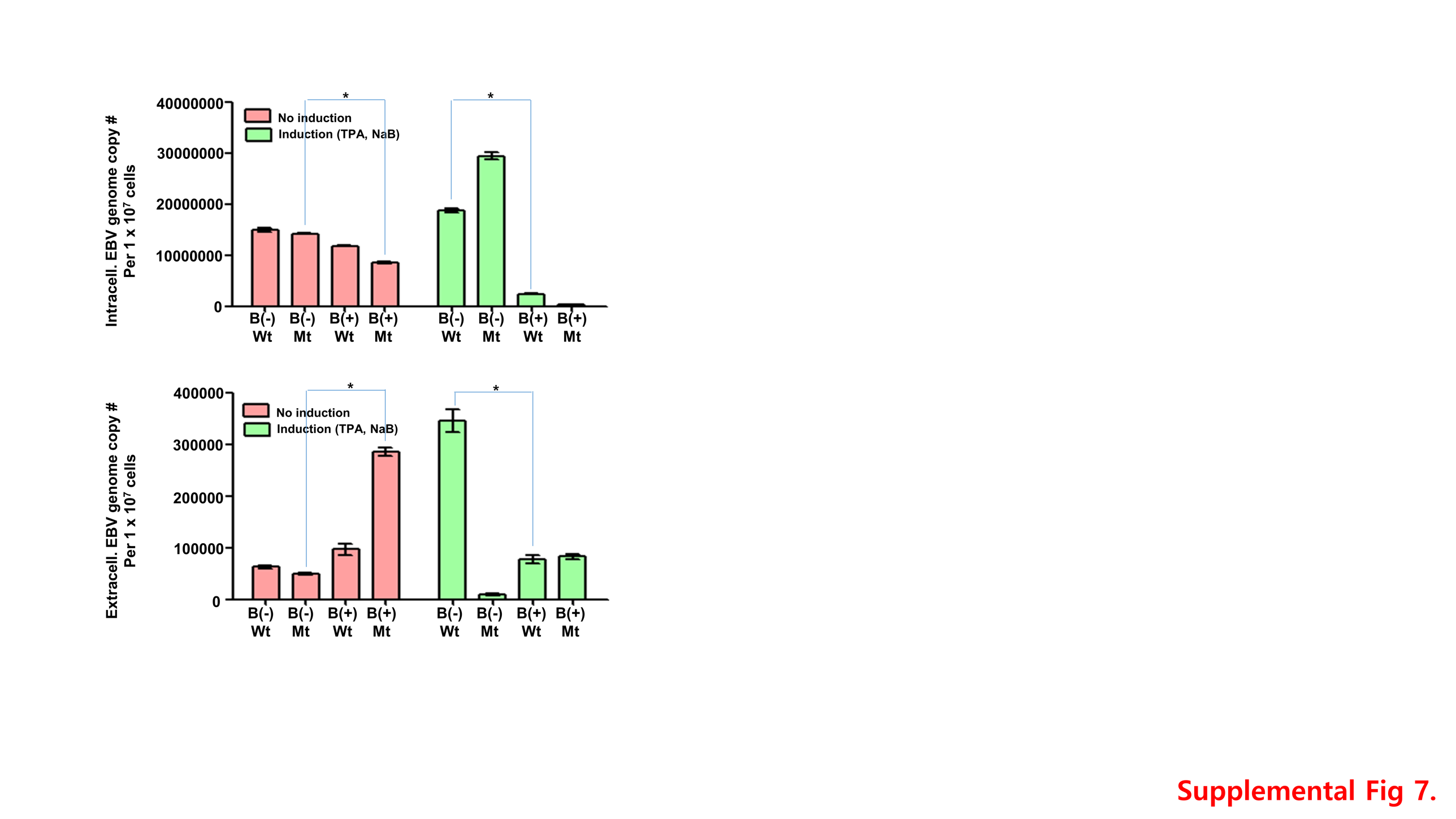

Supplement: S7 Fig — Absolute quantification of relative intracellular and extracellular EBV genome copy numbers. qPCR assay with EBNA1 primer set (EBV 96778–96979, EBV 96827–96845) was conducted to measure EBV genome copy numbers in all four types of HEK293-EBV cells which were either uninduced or induced with TPA and NaB. B(-)Wt, B(-)Mt, B(+)Wt, and B(+)Mt stand for HEK293-EBV cells of BART(-)·S13+, BART(-)·S13-, BART(+)·S13+, BART(+)·S13-, respectively. Both absolute intracellular and extracellular EBV genome copy numbers were evaluated based EBNA1 Ct values. Relation equation between absolute DNA amounts and EBNA1 Ct values was first defined and then applied to calculate DNA concentration (μg/μL) per length of template (bp) using a website called Copy Number Calculator of Technology NetWorks (https://www.technologynetworks.com/tn/tools/copynumbercalculator). In panels, experiments were independently repeated two times, and data are represented as mean ± SD. Statistical analysis was performed using both Kruskal-Wallis test (nonparametric test) as prior test and Dunn’s test as post test to compare among phenotypes of subject cells. (TIF) [file ppat.1011078.s007.TIF]

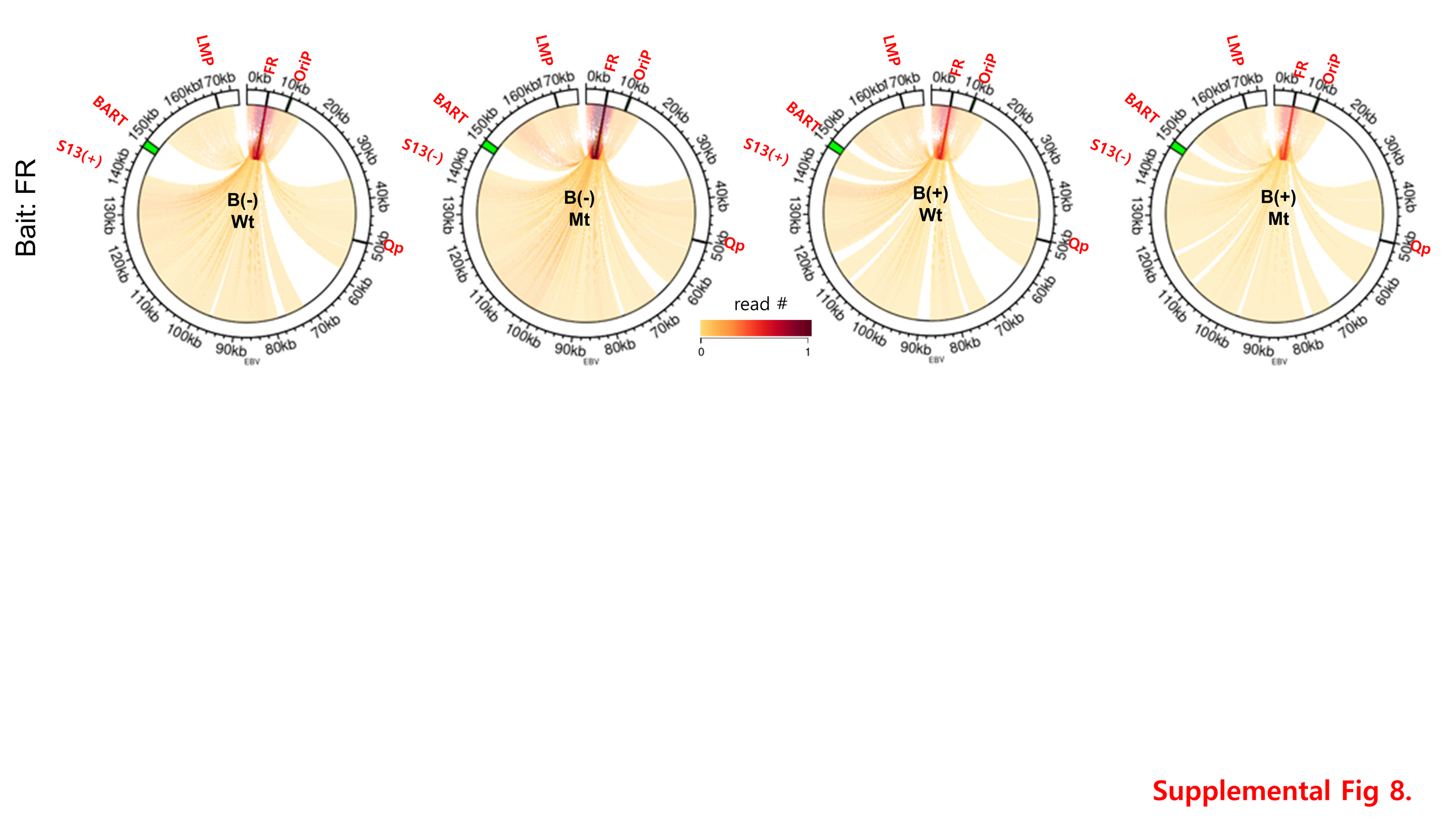

Supplement: S8 Fig — 4C-seq assays revealed all interactions of FR regions with other loci in EBV genomes in BART(+/-)·S13 HEK293-EBV cells. The FR viewpoint primer sets were located at EBV 4731–4751 and EBV 4941–4961. (TIF) [file ppat.1011078.s008.TIF]

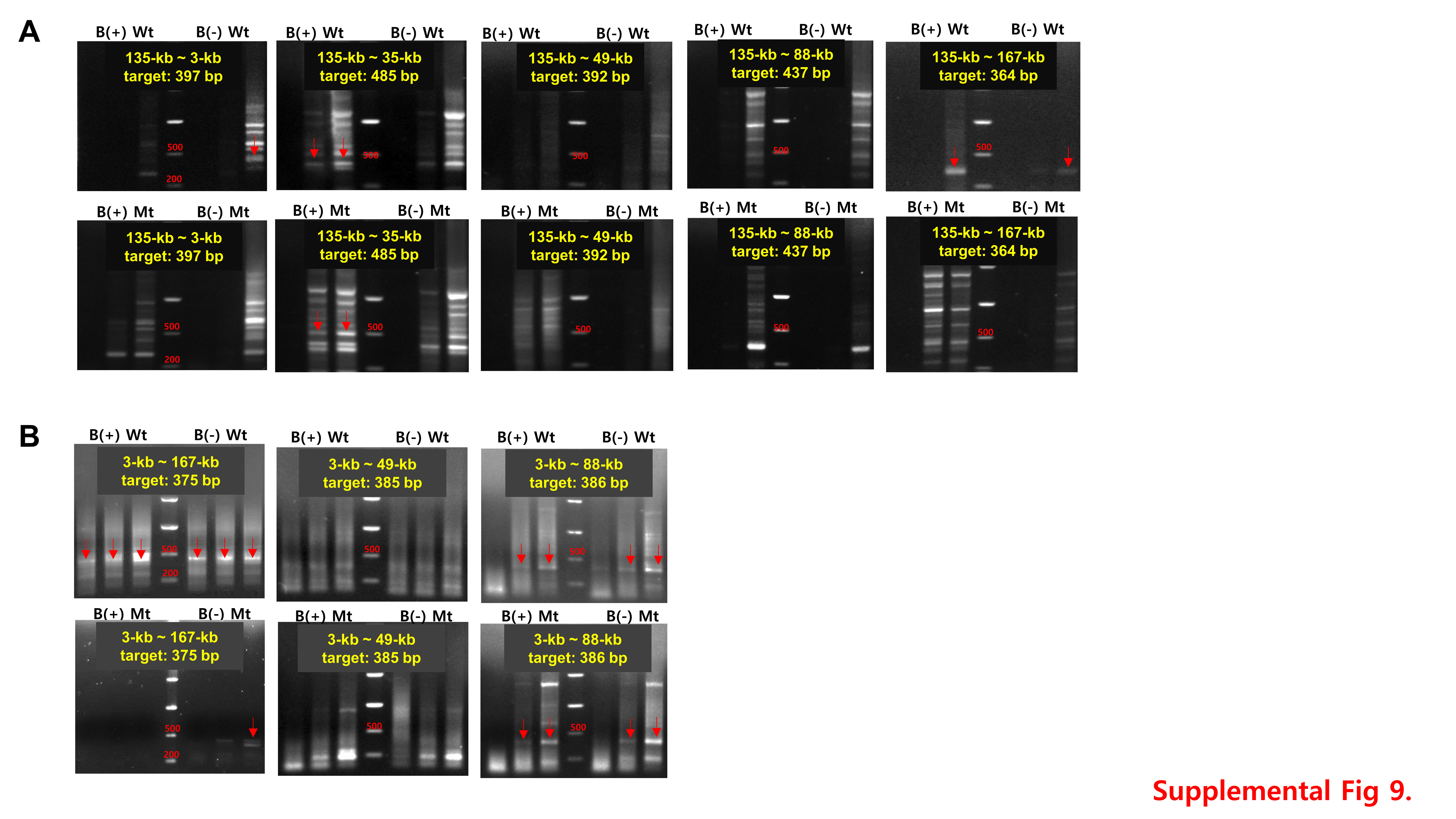

Supplement: S9 Fig — To verify EBV genomic associations defined by 4C-seq analysis, 3C-PCR assay was conducted using the 135-kb locus (F) and 3-kb locus (G) in Wt BART(+/-)·S13+ and Mt BART(+/-)·S13- HEK293-EBV cells. Linked associations between the bait and target regions were amplified as PCR products in the 3C-PCR assay. 3C DNA products were prepared and subjected to PCR assay using 0.5 μg (label-1), 5 μg (label-2), and 50 μg (label-3) of 3C DNA products as template to determine DNA associations within EBV genomic loci. The bait region was the 135-kb locus adjacent to S13. Target region sizes were 3-kb (near S1), 35-kb (near S3), 49-kb (near S5), 88-kb (near S11), and 167-kb (near S16), respectively. The tiny arrow indicates the PCR product suggesting association between the 135-kb region and one of the target regions in the 3C-PCR assay. 3-kb & 167-kb association was tested by 3C-PCR assay with OHK649 and OHK648 primer set, 135-kb & 3-kb association with OHK728 and OHK649 primer set, 135-kb & 35-kb association with OHK728 and OHK687 primer set, 135-kb & 49-kb association with OHK728 and OHK683 primer set, 135-kb & 65-kb association with OHK728 and OHK689 primer set, 135-kb & 88-kb association with OHK728 and OHK691 primer set, and 135-kb & 167-kb association with OHK728 and OHK648 primer set. (TIF) [file ppat.1011078.s009.TIF]

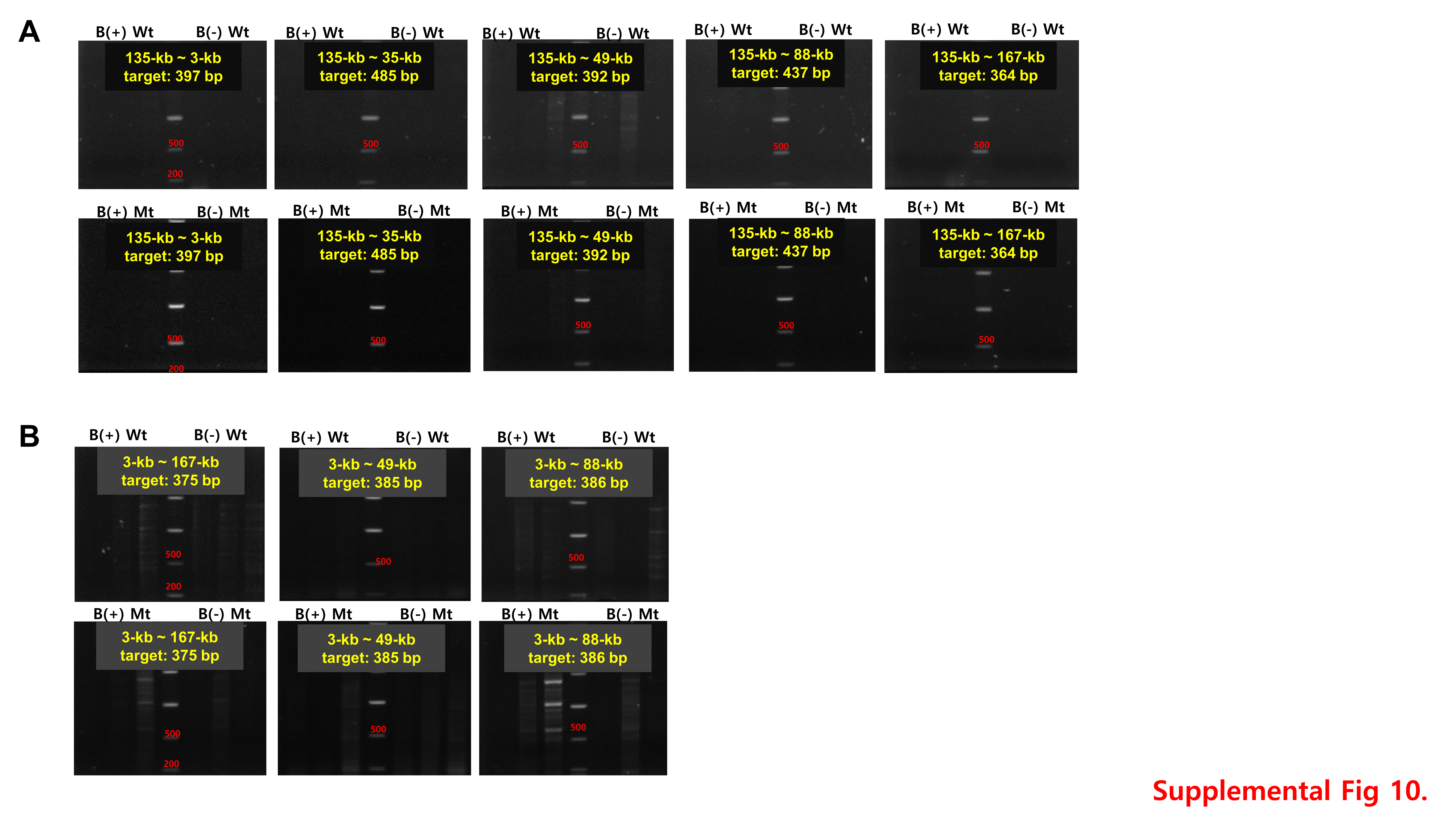

Supplement: S10 Fig — Negative experiments in 3C-PCR assay for analysis of EBV genomic associations were conducted using unligated XhoI-digested BART(+/-)·S13 HEK293-EBV DNA samples under the same conditions as the PCR assay with ligated XhoI-digested BART(+/-)·S13 HEK293-EBV DNA samples. Negative control experiments in 3C-PCR assay were conducted using the 135-kb locus (A) and 3-kb locus (B) in Wt BART(+/-)·S13+ and Mt BART(+/-)·S13- HEK293-EBV cells. Unligated XhoI-digested BART(+/-)·S13 HEK293-EBV DNA samples were not subjected to T4 DNA ligase mediated ligations. Unligated DNA samples were subjected to PCR assay using 0.5 μg (label-1), 5 μg (label-2), and 50 μg (label-3) of unligated DNA samples as template to determine false-positive amplification from primer sets in 3C-PCR assays. PCR primer sets used in analyzing ligated DNA samples were equally used exploited to negative experiments using unligated DNA samples. This PCR assay with unligated DNA samples were considered as negative control experiment to assess false positivity of 3C-PCR assay with ligated DNA samples. (TIF) [file ppat.1011078.s010.TIF]
