# Supplementary material for: Characterization of a new CCCTC-binding factor binding site as a dual regulator of Epstein-Barr virus latent infection
Source: PLoS Pathog. 2023 Jan 25;19(1):e1011078. doi: 10.1371/journal.ppat.1011078 (PMC9876287; doi:10.1371/journal.ppat.1011078)
Supplement: S1 Table — (DOCX) [file ppat.1011078.s011.docx]

**S1 Table. Sequences identified by ChIP-Seq assay as CTCF binding site in EBV genomes**

| Site  name | Sequences of  CTCF binding sites | Location (NC_007605) | Reference &  Features |
| --- | --- | --- | --- |
| S1^*^ | ^**^gggggccataaagcccagggtgtaaaacaccgaccgcgccaccagatggcacacgtgggggaaatgagggttagcataggcaacccccgcctacacacca | 6451 ~ 6550 | - adjacent to 3′ end of BNRF1 |
| S2 | cttaacacctgcagcctacaaaagtacactagctgtttgctctattcgcc | 10,501 ~ 10,550 | - adjacent to 3′ end of BCRF1  - adjacent to 5′ end of EBNA1 |
| S3 | cttgtgttagtgctatgtaatgcgttgccgccaggtggcagcctgtttat | 35,951 ~ 36,000 | - adjacent to 3′ end of EBNA2 |
| S4 | ccttaaggtgcgccacccttcctccttccgttttaatggtagaataacct | 40,932 ~ 40,981 | - adjacent to 5′ end of BHLF1  - adjacent to 5′ end of BHRF1  - ^***^same as S14 |
| S5 | cactgagggagtgttccacagtaatgttgtctggtcgctagatggcgcgggtgaggccacgctttgcgaaaacgaaagtgcttgaaaaggcgcgggatag | 50,051 ~ 50,150 | - adjacent to 3′ end of BOLF1  - adjacent to 3′ end of BFRF3 |
| S6 | tctcctttaaggtgctgtgagtagcaaaattctgcaaggccactaggcgc | 55,351 ~ 55,400 | - adjacent to 5′ end of BORF1 |
| S7 | catcacccctgtgggccccctggtggagaatataaagagcacctatctga | 63,501 ~ 63,550 | - adjacent to 5′ end of BORF2 |
| S8 | acaagcggccacaagggggttcccggcctgaatttgttaagctcactatg | 67,951 ~ 68,000 | - located in the middle of BMRF1 |
| S9 | Aaaggttccctcgttgaagcactgtgcgtgggccaaatagacgtagcgca | 73,751 ~ 73,800 | - located in the middle of BSLF1 |
| S10 | tcaggctgacgtccaccccaccccggcgccacctgcagggcttcactaga | 75,601 ~ 75,650 | - located in the middle of BLLF3 |
| S11 | ggccacgagggggcgggtgctcaggtccatctgtccacatatggctgct | 91,251 ~ 91,300 | - located in the middle of BRLF1 |
| S12 | ccagtgacgaagacccatctgagggcagtgatagcgacccctcgtggcat | 99,051 ~ 99,100 | - located in the middle of BKRF4 |
| S13 | atgcaagtgcatctttctaaccagtaggggcctccacctaggtgctttgttaatctttagtgggaactagtgggagtgctgtgcctcgggtacc | 138,901 ~ 138,994 | - adjacent to 5′ end of RPMS1 |
| S14 | ccttaaggtgcgccacccttcctccttccgttttaatggtagaataacct | 143,851 ~ 143,900 | - located in the middle of RPMS1  - ^***^same as S4 |
| S15 | gggcgcgagtagcacatggtctcggagccagggaccctcatgctcttgcg | 157,201 ~ 157,250 | - located in the middle of BALF4 |
| S16 | agaggaaggtaagagtgccatctatctgtacttttatttattgcatcacaagtcacatcaataataagggcgccatctagcgggagatgttatccacacc | 166,451 ~ 166,550 | - adjacent to 5′ end of LMP-2A |

^*^S1-16 sites are marked on Fig 1A.

^**^Sequences are CTCF binding sites defined by CTCF ChIP-Seq assay. The Integrative Genomics Viewer was applied to the CTCF ChIP-Seq bigwig file (sample) and EBV NC_007605 genome fastq file (reference). Higher peak areas were selected from S1 to S16, the sequences of which were selected from the EBV NC_007605 genome and are listed in S1 Table.

^**^S4 and S14 were identical in their sequences.
