# Supplementary material for: Characterization of a new CCCTC-binding factor binding site as a dual regulator of Epstein-Barr virus latent infection
Source: PLoS Pathog. 2023 Jan 25;19(1):e1011078. doi: 10.1371/journal.ppat.1011078 (PMC9876287; doi:10.1371/journal.ppat.1011078)
Supplement: S2 Table — (DOCX) [file ppat.1011078.s012.docx]

**S2 Table. Locations and sequences of primer sets used in ChIP-qPCR assay**

| Site  name | Primer name | Sequences | Locations  (NC_007605) |
| --- | --- | --- | --- |
| S1 binding site  primer set | F^**^: OHK133  R^***^: OHK134 | F: GCCACCAGATGGCACACGTG  R: GAGGTTTTGCAGTGTCTACA | F: 6488 ~ 6507  R: 6609 ~ 6628 |
| S2 binding site  primer set | F: OHK546  R: OHK547 | F: ACATTTTGTTCTAGGTCCATCTTAGGA  R: TGCCCTCCCCACTTCTCTT | F: 10411 ~ 10437  R: 10469 ~ 10487 |
| S3 binding site  primer set | F: OHK956  R: OHK957 | F: TGTGTGTTGTTGGCTTGTGT  R: GGGTGGAGGGAAAGGGATAC | F: 35938 ~ 35957  R: 36074 ~ 36093 |
| iC^*^ binding site  primer set | F: OHK548  R: OHK549 | F: GTAAGAAGCATGGCGAAGTAGA  R: TGCCCATGGAATGCTCAG | F: 38173 ~ 38194  R: 38255 ~ 38272 |
| S5 binding site  primer set | F: OHK642  R: OHK643 | F: AAATTGGGTGACCACTGAGGGAGT  R: ATAGCATGTATTACCCGCCATCCG | F: 50039 ~ 50062  R:50161 ~ 50184 |
| S8 binding site  primer set | F: OHK950  R: OHK951 | F: CCGTGTCATTTAGAAACCTTG  R: GTGAGCTTAACAAATTCAGGC | F: 67858 ~ 67878  R: 67976 ~ 67996 |
| S11 binding site  primer set | F: OHK958  R: OHK959 | F: TGTTGTGGTCAGTTCGTCCA  R: GAAGGCCCTAAGGGAGATGG | F: 91228 ~ 91247  R: 91329 ~ 91348 |
| S13 binding site  primer set | F: OHK536  R: OHK537 | F: GTGTCCGGTGGTCCTCACAC  R: GGTGGTGTGTGTTTACAGGG | F: 138844~138863  R: 139036~139055 |
| miR binding site primer set | F: OHK1005  R: OHK1006 | F: AAGTGACGTGCTGTGAATAC  R: GGGTCGTGACTATATAGGGG | F: 139360~139379  R: 139455~139436 |
| S15 binding site  primer set | F: OHK948  R: OHK949 | F: GGCACACCTCCGTCATCTTT  R: GCTCCGAGACCATGTGCTAC | F: 157100~157119  R: 157209~157228 |
| S16 binding site  primer set | F: OHK544  R: OHK545 | F: TATACGAAGAAGCGGGCAGAGGAA  R: TGACCTGTTGTCCCTGAGATGTGA | F: 166434~166457  R: 166559~166582 |

^*^iC represents internal control.

^**^F stands for forward direction.

^*^R represents the reverse direction.
