# Supplementary material for: Characterization of a new CCCTC-binding factor binding site as a dual regulator of Epstein-Barr virus latent infection
Source: PLoS Pathog. 2023 Jan 25;19(1):e1011078. doi: 10.1371/journal.ppat.1011078 (PMC9876287; doi:10.1371/journal.ppat.1011078)
Supplement: S3 Table — (DOCX) [file ppat.1011078.s013.docx]

**S3 Table. Locations and sequences of viewpoint primer sets used in 4C-Seq assay**

| Site name  (Viewpoint target) | Primer name | Sequences | Locations  (NC_007605) |
| --- | --- | --- | --- |
| OriP-*Mbo*I^*^ | ^**^R: OKK0181 | R: GGCATGACTTCCTCCAGGTAGAA | R: 9918 ~ 9940 |
| OriP-*Csp*6I | ^***^F: OKK0182 | F: GATTGCCTCTTGTGTTCTTGCC | F: 10281 ~ 10302 |
| LMP1/2-*Mbo*I | F: OKK0185 | F: CCTCCTCCCAACGCGTTTCT | F: 166661 ~ 166480 |
| LMP1/2-*Csp*6I | R: OKK0186 | R: GGCGCCCTTATTATTGATGTGACT | R: 166501 ~ 166524 |
| FR-*Mbo*I | R: OKK0187 | R: CGTGCATGGACCGGTTAATCC | R: 4731 ~ 4751 |
| FR-*Csp*6I | F: OKK0188 | F: TCTTTCGCGAGGTTAGGGACA | F: 4941 ~ 4961 |
| BART1-*Mbo*I | F: OKK0189 | F: CACACCTTTGAGGACACCTGG | F: 147470 ~ 147490 |
| BART1-*Csp*6I | R: OKK0190 | R: ACAGCCAACTCCATGGTTATGT | R: 147357 ~ 147378 |
| BART2-*Mbo*I | R: OKK0191 | R: GCTCCCTACCGAGGAAGGAT | R: 145801 ~ 145820 |
| BART2-*Csp6*I | F: OKK0192 | F: AAGTTTGGGCTTCGTCCCAG | F: 146004 ~ 146023 |
| Qp-*Mbo*I | F: OKK703 | F: GTATCCAACCGGCAGTGGAG | F: 51560 ~ 51579 |
| Qp-*Csp*6I | R: OKK704 | R: CGAGGTGCGCCTATCCC | R: 50278 ~ 50294 |
| S13-*Mbo*I | R: OKK762 | R: GCAAAACTCGGTTTACATACCCTGA | R: 138504 ~ 138528 |
| S13-*Csp*6I | F: OKK761 | F: GTGGGAGTGCTGTGCCTC | F: 138970 ~ 138987 |

^*^*Mbo*I primer is one of the viewpoint primers used in 4C-Seq to identify DNA associations with the OriP region.

^**^F stands for forward direction.

^*^R represents reverse direction.
