# Supplementary material for: Characterization of a new CCCTC-binding factor binding site as a dual regulator of Epstein-Barr virus latent infection
Source: PLoS Pathog. 2023 Jan 25;19(1):e1011078. doi: 10.1371/journal.ppat.1011078 (PMC9876287; doi:10.1371/journal.ppat.1011078)
Supplement: S4 Table — (DOCX) [file ppat.1011078.s014.docx]

**S4 Table. Adjustment of filtrated 4C-sequencing reads.**

| **4C-seq read numbers before adjusting (raw data)** | | | | | | |
| --- | --- | --- | --- | --- | --- | --- |
| **Read target**  **(viewpoint)** | **SNU719** | **B95-8** | **HEK293/**  **BART(+)·S13^+^** | **HEK293/**  **BART(+)·S13^-^** | **HEK293/**  **BART(-)·S13^+^** | **HEK293/**  **BART(-)·S13^-^** |
| **BART1** | 3,113,253 | 2 | 3,997,676 | 12,588,864 | 9,828 | 8,730 |
| **BART2** | 1,853,801 | 26 | 235,704 | 1,313,598 | 896 | 776 |
| **FR** | 592,763 | 12,805,441 | 639,840 | 2,231,928 | 769,826 | 2,047,194 |
| **LMP1/2** | 10,719 | 138,454 | 4,140 | 6,980 | 3,624 | 4,380 |
| **OriP** | 8,407,423 | 50,209,788 | 2,145,252 | 10,370,368 | 6,689,628 | 6,309,032 |
| **Qp** | 374 | 8,960 | 28 | 66 | 20 | 10 |
| **Total** | 13,978,333 | 63,162,671 | 7,022,640 | 26,511,804 | 7,473,822 | 8,370,122 |

| **4C-seq read numbers after adjusting to 7,000,000** | | | | | | |
| --- | --- | --- | --- | --- | --- | --- |
| **Read target**  **(viewpoint)** | **SNU719** | **B95-8** | **HEK293/**  **BART(+)·S13^+^** | **HEK293/**  **BART(+)·S13^-^** | **HEK293/**  **BART(-)·S13^+^** | **HEK293/**  **BART(-)·S13^-^** |
| **BART1** | 1,559,040 | 0 | 3,984,789 | 3,323,880 | 9,205 | 7,301 |
| **BART2** | 928,338 | 2 | 234,944 | 346,834 | 839 | 648 |
| **FR** | 296,840 | 1,419,163 | 637,777 | 589,304 | 721,021 | 1,712,085 |
| **LMP1/2** | 5,367 | 15,345 | 4,126 | 1,842 | 3,394 | 3,664 |
| **OriP** | 4,210,228 | 5,564,498 | 2,138,337 | 2,738,123 | 6,265,523 | 5,276,294 |
| **Qp** | 187 | 992 | 27 | 17 | 18 | 8 |
| **Total** | 7,000,000 | 7,000,000 | 7,000,000 | 7,000,000 | 7,000,000 | 7,000,000 |
