# Supplementary material for: Characterization of a new CCCTC-binding factor binding site as a dual regulator of Epstein-Barr virus latent infection
Source: PLoS Pathog. 2023 Jan 25;19(1):e1011078. doi: 10.1371/journal.ppat.1011078 (PMC9876287; doi:10.1371/journal.ppat.1011078)
Supplement: S5 Table — (DOCX) [file ppat.1011078.s015.docx]

**S5 Table. Adjustment of filtrated 4C-sequencing reads for S13 locus.**

| **Samples** | **Read target** | **filtered reads** | **removed duplicated reads** | **Adjusted final reads** |
| --- | --- | --- | --- | --- |
| **SNU719-bio1** | S13 | 4,331,088 | 7,226 | 3,000 |
| **SNU719-bio2** | S13 | 4,115,764 | 7,650 | 3,000 |
| **B95-8** | S13 | 20,967,080 | 40,256 | 3,000 |
| **HEK293/**  **BART(+)·S13^+^** | S13 | 1,758,822 | 4,504 | 3,000 |
| **HEK293/**  **BART(+)·S13^-^** | S13 | 1,252,158 | 3,456 | 3,000 |
| **HEK293/**  **BART(-)·S13^+^** | S13 | 7,350,274 | 7,002 | 3,000 |
| **HEK293/**  **BART(-)·S13^-^** | S13 | 230,892 | 3,874 | 3,000 |
