# Supplementary material for: Characterization of a new CCCTC-binding factor binding site as a dual regulator of Epstein-Barr virus latent infection
Source: PLoS Pathog. 2023 Jan 25;19(1):e1011078. doi: 10.1371/journal.ppat.1011078 (PMC9876287; doi:10.1371/journal.ppat.1011078)
Supplement: S6 Table — (DOCX) [file ppat.1011078.s016.docx]

**S6 Table. Locations and sequences of primer sets used in 3C-PCR assay**

| Site name | Primer name | Sequences | Locations  (NC_007605) |
| --- | --- | --- | --- |
| ^*^3-kb primer for S1 locus | R: OHK649 | R: ACAAACTGCTGCATTCCAGG | R: 3203 ~  3222 |
| 35-kb primer for S3 locus | R: OHK687 | R: AGGGGCTGGGGCTAAAAATG | R: 35519 ~  35538 |
| 49-kb primer for S5 locus | R: OHK683 | R: TATCAGGGAGGTGGTGACAG | R: 50002 ~  50021 |
| 65-kb primer for S8 locus | R: OHK689 | R: GGGATCCCTGAAGAATGGAG | R: 65534 ~  65553 |
| 88-kb primer for S11 locus | R: OHK691 | R: GTACAGTTCCAGTCTCACAC | R: 89214 ~ 89233 |
| 135-kb primer for S13 locus | R: OHK728 | R: CTTCAGGACCCACGCAAGGTC | R: 136109 ~ 136129 |
| 167-kb primer for S16 locus | F: OHK648 | F: AGGCATAAAAGTCCAAACAG | F: 166859 ~ 166878 |

^*^ 3 K primer is one of the viewpoint primers used in 3C-PCR to identify DNA association with the 3-kb region in the EBV NC_007605 genome.

^**^F stands for reverse direction.

^***^R stands for forward direction.
