# Supplementary material for: Characterization of a new CCCTC-binding factor binding site as a dual regulator of Epstein-Barr virus latent infection
Source: PLoS Pathog. 2023 Jan 25;19(1):e1011078. doi: 10.1371/journal.ppat.1011078 (PMC9876287; doi:10.1371/journal.ppat.1011078)
Supplement: S7 Table — (DOCX) [file ppat.1011078.s017.docx]

**S7 Table. Quantification of band intensity by densitometry* of Southern blot analysis**

| EBV genomes | Top band  (over 8.5-kb) | Middle band  (= 8.5-kb) | Bottom band  (= 5.0-kb) |
| --- | --- | --- | --- |
| B(-) Wt 10** | 223.6*** (153, 249****) | 221.6 (136, 249) | 158.4 (0, 249) |
| B(-) Wt 20 | 203.0 (119, 249) | 196.2 (68, 249) | 111.1 (0. 249) |
| B(-) Mt 10 | 245.2 (238, 249) | 243.5 (238, 249) | 198.2 (0, 249) |
| B(-) Mt 20 | 243.8 (238, 249) | 239.5 (221, 249) | 137.0 (0, 249) |
| B(+) Wt 10** | 218.2 (129, 249) | 231.6 (168, 249) | 216.3 (135, 249) |
| B(+) Wt 20 | 174.0 (0, 249) | 216.4 (66, 249) | 180.0 (0, 249) |
| B(+) Mt 10 | 243.5 (230, 249) | 243.0 (222, 249) | 204.1 (19, 249) |
| B(+) Mt 10 | 241.0 (200, 249) | 238.3 (184, 249) | 160.1 (0, 249) |

^*^ ImageJ was used to quantify band intensity on Southern blot data.

^**^For all bands, rectangle area are 756 (B(-) sample) and 748 (B(+) sample).

^***^Mean intensity is listed in blue.

****Minimum intensity (0 means dark band) and maximum intensity (249 mean white bands) are listed in parenthesis.
