# Supplementary material for: Characterization of a new CCCTC-binding factor binding site as a dual regulator of Epstein-Barr virus latent infection
Source: PLoS Pathog. 2023 Jan 25;19(1):e1011078. doi: 10.1371/journal.ppat.1011078 (PMC9876287; doi:10.1371/journal.ppat.1011078)
Supplement: Attachment — Submitted filename: Responses to Reviwers comments_1st revision.docx [file ppat.1011078.s018.docx]

Dear Dr. Kang,

Thank you very much for submitting your manuscript "Characterization of a new CCCTC-binding factor binding site as a dual regulator of Epstein-Barr virus latent infection" for consideration at PLOS Pathogens. As with all papers reviewed by the journal, your manuscript was reviewed by members of the editorial board and by several independent reviewers. In light of the reviews (below this email), we would like to invite the resubmission of a significantly-revised version that takes into account the reviewers' comments.

Thank you for sharing your thorough experiments studying the role of CTCF binding in regulating EBV chromatin and transcription. The careful reviewers have noted many questions and suggestions regarding the arguments presented. The most important point of improvement for the revision is an increased focus on rigor and reproducibility. Please clarify concerns about statistics, technical vs. biological replicates, data presentation, and use of the appropriate controls. If any critical elements are missing, they must be included in the revision. These critiques particularly, though not exclusively, apply to the Western blot, 4C-seq, ChIP-qPCR, and 3C-PCR.

We cannot make any decision about publication until we have seen the revised manuscript and your response to the reviewers' comments. Your revised manuscript is also likely to be sent to reviewers for further evaluation.

When you are ready to resubmit, please upload the following:

[1] A letter containing a detailed list of your responses to the review comments and a description of the changes you have made in the manuscript. Please note while forming your response, if your article is accepted, you may have the opportunity to make the peer review history publicly available. The record will include editor decision letters (with reviews) and your responses to reviewer comments. If eligible, we will contact you to opt in or out.

[2] Two versions of the revised manuscript: one with either highlights or tracked changes denoting where the text has been changed; the other a clean version (uploaded as the manuscript file).

Important additional instructions are given below your reviewer comments.

Please prepare and submit your revised manuscript within 60 days. If you anticipate any delay, please let us know the expected resubmission date by replying to this email. Please note that revised manuscripts received after the 60-day due date may require evaluation and peer review similar to newly submitted manuscripts.

Thank you again for your submission. We hope that our editorial process has been constructive so far, and we welcome your feedback at any time. Please don't hesitate to contact us if you have any questions or comments.

Sincerely,

JJ Miranda
Guest Editor
PLOS Pathogens

Erik Flemington
Section Editor
PLOS Pathogens

Kasturi Haldar
Editor-in-Chief
PLOS Pathogens
orcid.org/0000-0001-5065-158X

Michael Malim
Editor-in-Chief
PLOS Pathogens
orcid.org/0000-0002-7699-2064

***********************

Thank you for sharing your thorough experiments studying the role of CTCF binding in regulating EBV chromatin and transcription. The careful reviewers have noted many questions and suggestions regarding the arguments presented. The most important point of improvement for the revision is an increased focus on rigor and reproducibility. Please clarify concerns about statistics, technical vs. biological replicates, data presentation, and use of the appropriate controls. If any critical elements are missing, they must be included in the revision. These critiques particularly, though not exclusively, apply to the Western blot, 4C-seq, ChIP-qPCR, and 3C-PCR.


Reviewer's Responses to Questions

**Part I - Summary**
Please use this section to discuss strengths/weaknesses of study, novelty/significance, general execution and scholarship.

Reviewer #1: This manuscript describes the interaction of chromatin insulator CTCF with EBV episomes and identifies a novel CTCF binding site in the BART miRNA promoter. The authors go on to characterize the function of this CTCF binding site by 1. comparing EBV isolates that with and without a deletion in the region of the novel CTCF binding site and 2. by site directed mutagenesis of EBV bacmids to ablate CTCF binding. The findings are of interest and timely and the study has the potential to add new knowledge to that already reported. However, there are several technical issues (highlighted below) that need to be resolved. In addition, the manuscript is poorly written in parts and therefore difficult to follow. In my opinion, the manuscript cannot be accepted for publication in it's current state.

Reviewer #2: In this manuscript the authors identified CTCF binding site on the EBV genome in two different EBV+ cell lines that carry different EBV genomes. While SNU719 EBV is intact, there is a 11.8kb deletion near to S13 CTCF binding site in B95-8 EBV. The authors used 4C-seq, 3C-PCRs and EBV mutagenesis to explore the biological role of CTCF binding in the BART miRNA promoter region (S13 locus) regarding EBV gene regulation. The results suggest that the CTCF binding in S13 locus reduces RNA polymerase II binding at S13 and also reduces EBV gene expression. In contrast, mutation of CTCF-binding site in S13 reduces EBV infectivity suggesting that CTCF in S13 locus may be required for EBV replication, or virus production or infection. While the manuscript includes a large amount of work and data, it requires some additional experiments in some cases to support their conclusion or to clarify ambiguous statements.

Reviewer #3: CTCF insulators are important transcriptional regulators in eukaryotic cells and it is becoming increasingly clear that these insulators-together with cohesin- play important roles in controlling transcription of DNA viruses through the formation of 3D chromatin loops, including in EBV. In this current work, Lee et al expand on the current knowledge of 3D loops in EBV by identifying additional long-range interactions between sequences mapping near the BART region of EBV and the S13 locus. These data suggest that the S13 locus is involved in BART miRNA expression. The data presented by Lee et al are extensive. For example, they perform ChIP seq experiments to show that 16 sites in both B95-8 and SNU719 genomes are enriched in CTCF, they show biological function through functional assays (conventional ChIP, gene expression, mutational analyses, etc) and showed that mutations in S13 resulted in decreased integration into cellular genomes, and a reduction in EBV infectivity. There are a few areas of the manuscript that are more descriptive than others. For example, it would be nice to describe mechanistically why CTCF, RNA Pol II, and histones are more enriched at S13 in B95-8 and not SNU719. What effect does this have on the viral phenotype? Viral fitness? replication? reactivation? Finally, while the data is compelling, I do not think that the authors truly show that the long-range interaction coordinates BART miRNA expression and infectivity- more mechanistic evaluations in cell types other than HEK cells should be done to definitively prove this. My one major concern with the manuscript is the absolute lack of detail in the number of replicates done for each experiment and the lack of statistics that identify what is statistically different. Specific comments are below:

**Part II – Major Issues: Key Experiments Required for Acceptance**
Please use this section to detail the key new experiments or modifications of existing experiments that should be absolutely required to validate study conclusions.

Generally, there should be no more than 3 such required experiments or major modifications for a "Major Revision" recommendation. If more than 3 experiments are necessary to validate the study conclusions, then you are encouraged to recommend "Reject".

“In revised manuscript, we marked in red color everywhere we revised in the old manuscript as reviewers’ comments.”

Reviewer #1:

**R#1-Comment-1-1:** Deletion in EBV genome in B95-8 should be annotated in **figure 1A**.

R#1-Response-1-1: We added annotations for 11.8-kb EcoRI C fragment, OriP, Qp, and LMP1/2 promoter (LMP1/2p) in new Figure 1A.

**R#1-Comment-1-2:** Annotation of key EBV genes and enhancer/promoter features in this figure would also be useful.

R#1-Response-1-2: We added annotations for 11.8-kb EcoRI C fragment, OriP, Qp, and LMP1/2 promoter (LMP1/2p) in new Figure 1A.

**R#1-Comment-1-3:** Coverage in the deleted region in B95-8 in the IgG ChIP-Seq track in SNU719 cells appears to be markedly reduced in the same region. How do the authors explain this when there is no deletion in this region in these cells?

R#1-Response-1-3: We repeated the IgG ChIP-Seq using SNU719 cells and continuously observed the reduction in the same SNU719 region corresponding to the deleted B95-8 region. Interestingly, we found similar reduction in the upstream region of S4, like the S14 upstream region. We carefully speculate that signal reduction in both regions might be due to the sequence duplicity between S4 and S14 upstream regions. The sequence duplicity might reduce read signals in the middle areas of S3 and S4, and S13 and S14 in IgG and CTCF ChIP-seq tracts.

**R#1-Comment-2-1: Fig2A** – it is impossible to tell the quality of the purified CTCF protein from the western blot provided for the following reasons: it is tightly cropped so that spurious bands cannot be seen (in fact there are multiple bands in the WB section that is shown), there is no molecular weight marker so the size of the protein is not indicated and, most importantly, the authors do not provide a Coomassie stained gel image to show a single, purified band.

R#1-Response-2-1: We repeated the CTCF western blot assay and posted the new data in new Figure 2A and new Supplemental Figure 6A (below).

We used Sf9 cell lysates in which CTCF was overexpressed in this assay. As control, we used Sf9 cell lysates in which any protein was not overexpressed. For reference, we posted the coomassie stained gel of lysates of Sf9 and Sf9 with CTCF overexpression in new Supplemental Figure 6B (below).

“Western blot data”	“Coomassie stained gel”

R#1-Comment-2-2: In **fig 2B** the authors show the mutations introduced into the S13 CTCF binding site. These are extensive mutations. Is this a coding region, or are there any other known features in this region of the EBV genome. How can the authors be sure they are not affected another viral function with such dramatic changes?

R#1-Response-2-2: *RPMS1* gene is in EBV genome 138352 ~ 160531. mRNA encoding RPMS1 are in EBV genome 138352 ~ 138480, EBV genome 149581 ~ 149712, EBV genome 149923 ~ 150077, EBV genome 150237 ~ 150348, EBV genome 155267 ~ 156737, EBV genome 156846 ~ 156928, and EBV genome 158625 ~ 160531. The mutated sequence in S13 is in EBV genome 138963 ~ 138987 and thus located in intron region of *RPMS1* gene. We speculated that the genetic mutation in intron region of RPMS1 gene may not cause a huge effect on EBV function.

R#1-Comment-2-3: They apparently show no change in **RPMS1 expression** in Fig S3B but this is just a sequence alignment, not expression data.

R#1-Response-2-3: To clear that the genetic mutation in S13 may not cause significant change in RPMS1 expression, we conducted qPCR assay to compare RPMS1 expression between S13 wild-type and S13 mutant HEK293-EBV cells. The qPCR assay was carried out with primer set (EBV genome 139360-139379, EBV genome 139455-139436) to quantify the *RPMS1* transcript. Amount of *RPMS1* transcript in BART(-)·S13^-^ HEK293-EBV cells was lower up to 33.3% than the amount of *RPMS1* transcript in BART(-)·S13^+^ HEK293-EBV cells. However, Amount of *RPMS1* transcript in BART(+)·S13^-^ HEK293-EBV cells was not different from the amount of *RPMS1* transcript in BART(-)·S13^+^ HEK293-EBV cells. These data are posted in new Supplemental Figure 4G.

R#1-Comment-2-4: What is the biological effect of the sequence differences between WT and mutant? This becomes most important in the interpretation of the infectivity assays shown in **Figure 8.**

R#1-Response-2-3: We expected that WT S13 is suitable for CTCF binding site but mutant S13 is not appropriate for CTCF binding site.

R#1-Comment-3-1: The labelling of **Fig 2C** is confusing. The top two panels are data collected following addition of Sf9 lysate (?) but the fluorescence traces still labelled with CTCF. The authors should calculate **Kd values** from these traces (ideally from multiple experiments +/- S.D.) rather than relying on the visual interpretation of the shift in the trace upon addition of protein.

R#1-Response-3-1: We calculated Kd values from these traces. Especially, we repeated MST experiments to compare the binding activity of CTCF to wild-type S13 oligo with the activity of Sf9 lysate to mutant S13 oligo. Resultant new data were posted in new Figure 2C.

Using these new data, we calculated Kd values for the two traces. Kd value of “wild-type S13 oligo + CTCF” trace was 4.8 ± 2.3 µM and the Kd value of “mutant S13 oligo + Sf9” trace was 6.5 ± 3.7 µM. We denoted the Kd values in Figure 2C. We also addressed the difference of Kd values in manuscript.

R#1-Comment-4-1: **Figure 5** needs to be explained more fully. Are Bio1 and Bio2 replicates?

R#1-Response-4-1: Yes, Bio1 and Bio2 are replicates. To avoid this confusion, we put one representative image instead of two biological replicates.

R#1-Comment-4-2: What do the numbers next to the legend for each Circos plot mean? Annotation of the location of the CTCF binding sites identified (S1-16) would be helpful for interpretation.

R#1-Response-4-3: We annotated the viewpoints and key features in each circos plot in new Figure 5.

R#1-Comment-5-1: The 3C experiments shown in **Fig5D** and Figure 9 would benefit from a figure explaining the method and location of primers used. What are the lanes labelled 1, 2 and 3.

R#1-Response-5-1: label 1, 2 and 3 indicates template amounts such as 0.5ng, 5ng, and 50ng used in 3C-PCR assay. We mentioned amount of template DNAs used in 3C-PCR assay in Method and Materials and figure legend (Fig5F, 5G) of manuscript (subtitled as “3C-PCR and 4C-Seq assays”).

R#1-Comment-5-2: There do not appear to be any positive or negative controls for this experiment.

R#1-Response-5-2: We used “DNA link between 3-kb and 167-kb” as “positive control” in this 3C-PCR assay. “DNA link between 3-kb and 167-kb” was reported in previous studies (reference: attached below). But we did not include any special negative control. 3C-PCR assay showed variable data in our hands. Actually, we observed slightly different results in each 3C-PCR assay and not such convincing these 3C-PCR data.

R#1-Comment-5-3: Were the products Sanger sequenced?

R#1-Response-5-3: We sequenced PCR products for “DNA link of 3-kb and 167-kb” and “DNA link of 167-kb and 135-kb”. Sequencing results were posted in new Supplemental Figure 2.

R#1-Comment-5-4: Why are there multiple bands visible for each experiment?

R#1-Response-5-4: We do not have answer why there were multiple bands in 3C-PCR assay. We carried out PCR assay in harsh condition whose annealing temperatures were high and template DNA amounts were low up to 5 ng. In spite of this harsh condition, we could observe multiple bands on some tests. To verify the linkage of anchor region to target region in EBV genome, we sequenced a few of the linked DNA fragments and confirmed them. However, we did not sequence all multiple bands. In the end, we wanted to use these data to partially support 4C-seq data.

R#1-Comment-6-1: **Figure 6B** – molecular weight markers should be labelled on the Southern blot.

R#1-Response-6-1: We added molecular weight markers on the Southern blot data in new Figure 6B.

R#1-Comment-6-2: To draw the conclusion stated in lines 286-288, the authors need to quantify band intensity by densitometry of several repetitions of the Southern blot analysis.

R#1-Response-6-2: We tried Southern blot assay five times and obtained the best result from fourth and fifth trials. After we got the best result, we did not try more the Southern blot assay. However, we quantified band intensity of the best Southern blot and posted in new Supplemental Table 6 (below).

|  | Top band (over 8.5-kb) | Middle band (= 8.5-kb) | Bottom band (= 5.0-kb) |
| --- | --- | --- | --- |
| B(-) Wt 10 | 223.6 (153, 249) | 221.6 (136, 249) | 158.4 (0, 249) |
| B(-) Wt 20 | 203.0 (119, 249) | 196.2 (68, 249) | 111.1 (0, 249) |
| B(-) Mt 10 | 245.2 (238, 249) | 243.5 (238, 249) | 198.2 (0, 249) |
| B(-) Wt 20 | 243.8 (238, 249) | 239.5 (221, 249) | 137.0 (0, 249) |
| B(+) Wt 10 | 218.2 (129, 249) | 231.6 (168, 249) | 216.3 (135, 249) |
| B(+) Wt 20 | 174.0 (0, 249) | 216.4 (66, 249) | 180.0 (0, 249) |
| B(+) Mt 10 | 243.5 (230, 249) | 243.0 (222, 249) | 204. 1 (19, 249) |
| B(+) Mt 20 | 241.0 (200, 249) | 238.3 (184, 249) | 160.1 (0, 249) |

- ImageJ was used to quantify band intensity on Southern blot data.

- For all bands, rectangle area are 756 (B(-) sample) and 748 (B(+) sample), mean intensity is listed on top, minimum intensity (0 means dark band) and maximum intensity (249 mean white bands) are listed in parenthesis.

R#1-Comment-7-1: It is unclear what the error bars indicate in the ChIP-qPCR experiments shown in **figures 3 and 7**. Is error calculated from multiple repetitions of the qPCR of a single ChIP experiment, or from the mean of multiple independent ChIP experiments.

R#1-Response-7-1: We did two independent ChIP experiments. Each ChIP experiment has three replicates of qPCR assay at test-points. We pool two independent experiment sets whose results have three repetitions of the qPCR per a single ChIP experiment (thus, we obtained six results and processed them statistically) and posted modifications in new Figure 3 and Figure 7 as recommended by reviewer #1.

I suspect they are the former as there are little variation in the data. If so, I am not confident that the differences observed between cell types and EBV bacmid genomes are meaningful.

R#1-Response-7-2: We performed three independent ChIP experiments and the differences observed between cell types and EBV bacmid genomes were showed similar patterns in three independent experiments. In addition, we presented several data obtained from different experiments. ChIP data are well consistent to miRNA expression data (Fig6C). Thus, the best result from multiple repetitions of the qPCR of a single ChIP experiment can be meaningful in this study.

These experiments should be repeated multiple times and reported as individual experiments in the supplementary or mean and error of independent experiments.

R#1-Response-7-3: As reviewer’s recommendation, we pooled all data which are composed of two independent ChIP experiment; Each ChIP experiment has three qPCR repetitions per measurement points. Then we posted these modifications in new Figure 3 and Figure 7 as recommended by reviewer #1.

R#1-Comment-8-1: What does **figure 8A** show? This panel does not add anything meaningful.
R#1-Response-8-1: We removed old Figure 8A and transferred into new Supplemental Figure 5.

R#1-Comment-9-1: **Lines 384-387**. How do the authors conclude that the interactions in EBV episomes are ‘simultaneous’. Presumably there are multiple copies of the viral episome in a single cell. They are analysing the episome conformations in the entire population. There may be a mixture of different forms that cannot be unpicked from the bulk population.

R#1-Response-9-1: Thanks for this insightful comment. We agree with reviewer’s comment, and ~~We~~ removed ‘simultaneous’ from our conclusion in manuscript.

Reviewer #2:

**R#2-Comment-1-1:** in **Fig 1,** CTCF ChIP-seq was done in two different cell lines but there is no mention in the manuscript about their comparison

R#2-Response-1-1: We mentioned their comparison in the “Results session” titled “Distribution of CTCF binding site in EBV genomes”.

**R#2-Comment-1-2:** or how much this data is aligned with previous CTCF ChIP-seq on EBV genome.

R#2-Response-1-2: We aligned 3.9 x 10^6^ CTCF ChIP reads and 2.7 x 10^6^ IgG ChIP reads for B95-8 cells, and 4.4 x 10^6^ CTCF ChIP reads and 3.2 x 10^6^ IgG ChIP reads for SNU719 cells.

**R#2-Comment-1-3:** What is Y axis on the graph?

R#2-Response-1-3: Y axis is ChIP reads on EBV genomes. Their reads were adjusted in group autoscale. We mentioned this annotation on the Y axis as indicated by Integrative Genomic Viewer.

**R#2-Comment-1-4:** Why is the sequence of S4 and S14 identical?

R#2-Response-1-4: EBV genome 40775-41250 is repeated with EBV genome 143694-144169. S4 and S14 are located at EBV genome 40775-41250 and EBV genome 143694-144169, respectively.

**R#2-Comment-1-5:** Line 132 Multiple Em…What is Em?

R#2-Response-1-5: MEME stands for “Multiple EM for Motif Elicitation” on the website (<https://meme-suite.org/meme/tools/meme>). Em is part of “Multiple EM for Motif Elicitation”.

**R#2-Comment-1-6:** How similar is the CTCF binding site logo to the consensus CTCF sites identified in previous studies?

R#2-Response-1-6: Based on simple comparison, the logo of CTCF binding sites defined in this study is similar to LowOc or MedOc models suggested in CTCF binding site reference paper (Kobby Essien’s et al. Genome Biology 2009, 10(11):R131). We mentioned this similarity in the “Results session” titled “Distribution of CTCF binding site in EBV genomes”.

Figure 1: Genome Biology 2009, 10(11):R131

Figure 2: Logo of CTCF binding sites defined in this study

**R#2-Comment-2-1:** in **Fig 2A,** MW is missing from the immunoblot.

R#2-Response-2-1: We put a MW for immunoblot in new Supplemental Fig 6.

**R#2-Comment-2-2:** Is this lysate or purified CTCF on the immunoblot?

R#2-Response-2-2: We used Sf9 cell lysates in which CTCF protein was overexpressed.

**R#2-Comment-3-1:** In **Fig 2C**, the bound oligo/CTCF curve should not be on the top while unbound should be the lower curve?

R#2-Response-3-1: Thanks for this comment. We changed locations of bound signal and unbound signal in MST curve in new Fig2C-F.

**R#2-Comment-3-1:** What is [-] on the Y axis?

R#2-Response-3-2: [-] means nothing.

**R#2-Comment-3-3:** What does the blue and pink vertical lines represent?

R#2-Response-3-3: Blue vertical and pink lines indicate time of initial fluorescence (no molecule flow) and time of thermophoresis/ backdiffusion (molecule flow), respectively. We added this information in figure legend (Fig2).

**R#2-Comment-3-4:** Their labels are missing.

R#2-Response-3-4: We added their labels.

**R#2-Comment-3-5:** It would be better to use different line colors for the different CTCF conc.

R#2-Response-3-5: We would like to use different line colors for the different CTCF conc. However, we could not change these original colors into different line colors due to lack of our skill to operate monolith.

**R#2-Comment-4-1:** **Fig 3.** The authors wanted to investigate the binding of epigenetic factors and RNAPII on the BART miRNA region (S13) but instead the graphs show the enrichment of different transcription-related factors at different CTCF binding site including S13. Can they test for more genomic sites around S13 spanning the BART miRNA promoter in more detail?

R#2-Response-4-1: We added two more genomic sites around S13 in new Fig 3. These are miR-BART (miR : EBV genome 139360-139379, 139455-139436) and S15 site (EBV genome 157100-157119, 157209-157228).

**R#2-Comment-4-2:** Also, I recommend that they add a BART miRNA promoter region schematic and indicate where the ChIP-qPCR primers are located.

R#2-Response-4-2: We added a BART miRNA promoter region in Figure 4A schematic and indicated where the miR ChIP-qPCR primer (EBV genome 139360-139379, 139455-139436) are located in Figure 4A schematic.

**R#2-Comment-5-1: Line 170**: delete the word histone after H3K4me3 and H3K9me3. They are histone marks.

R#2-Response-5-1: We appreciate this comment. We removed histone in the manuscript.

**R#2-Comment-6-1:** I agree with the observation that CTCF, RNAPII and H3K4me3 are more enriched at S13 and S16 in B95-8 than in SNU719. While the 11.8kb EcoRI fragment may play a role in this discrepancy but another possibility, which was not excluded, that quality of chromatin and thereby that of ChIPs are different.

R#2-Response-6-1: We appreciated your agreement on your argument about the role of 11.8-kb EcoRI fragment on making these discrepancy in distribution of CTCF, RNAPII and H3K4me3.

**R#2-Comment-7-1:** In fact, in **Fig 1A** CTCF ChIP-seq seems to have higher background or was less efficient in SNU719 compared to B95-8.

R#2-Response-7-1: We repeated CTCF ChIP-seq one more time and still found that higher background in SNU719 compared to B95-8. We repeated at least three time CTCF ChIP-seq assay using SNU719 cells and found high backgrounds.

**R#2-Comment-8-1: Line 180-182**: I do not see how the ChIP data in Fig 3 indicate that S13 locus plays a role in the expression of BART miRNAs by coordinating the enrichment of RNAPII, CTCF, and histones. Why histones? No histones were measured here. In addition, there was no functional analysis, and EBV is defective near to S13 in B95-8. Their conclusion would be valid if the 11.8kb fragment were added back to the EBV genome in B95-8 and they would see different BART miRNA expression and changes in RNAPII and in the enrichment of histone mark.

R#2-Response-8-1: We accepted reviewer’s concerns about the lack of functional study about S13 locus using B95-8 and SNU719 cells. Thus, we only mentioned the potential role of S13 locus in expressing BART miRNAs (is 🡺 might be).

**R#2-Comment-9-1: Fig 4**. Is the viral gene expression difference between B95-8 and SNU719 restricted to BART miRNAs or there is difference in the expression of other EBV genes as well?

R#2-Response-9-1: We compared other EBV gene expression difference between B95-8 and SNU719 cells except BART miRNAs. The comparison in EBV gene expression was posted in new Figure 4D, 4E.

**R#2-Comment-10-1: Fig 5** is supposed to show chromatin interaction of S13 locus with other parts of the EBV genome. Instead, the 4C-seq shows the interactions of 8-9kb upstream BART regions with the EBV genome. Although the data is interesting but it is unclear why they did not use S13 locus as a viewpoint for the 4C-seq?

R#2-Response-10-1: We appreciate this insightful comment. As reviewer recommended, ~~However,~~ we conducted another 4C-seq analysis using S13 locus as a viewpoint with B95-8, SNU719-bio1, SNU719-bio2, and four type of HEK293 cells posted their data in new Fig5A, 9A, Sup Figure 1C, and Sup Figure 7B.

We adapted BART region (EBV genome 147357~147490) as a 4C-seq bait because it could work internal control in 4C-seq assay applied to B95-8 and SNU719 cells. EBV in B95-8 cells has a deletion of BART bait but EBV in SNU719 cells contains the full length of BART bait. As shown in new Figure 5A and new Figure 9A, In 4C-seq assay utilizing BART bait, there were not signals in samples from B95-8 cells and BART(-)·S13 HEK293-EBV cells..

**R#2-Comment-11-1:** In **Fig 5D** some of the 3C-PCRs do not seem specific but generate several bands. How do we know which band corresponds to what specific DNA sequence?

R#2-Response-11-1: We sequenced “linked DNA fragments” such as 3-kb ~ 135-kb, 167-kb ~ 135-kb, and “3-kb ~ 167-kb”. Sequence data for 3-kb ~ 135-kb and 167-kb ~ 135-kb linked DNA fragments were posted in new Supplemental data 6. However, the sequence data for 3-kb ~ 167-kb linked DNA fragment was used for only reference control and not posted.

**R#2-Comment-12-1: Line 262.** The bacmid DNAs were transfected and not transduced into HEK293.

R#2-Response-12-1: Thanks for this comment. We fixed manuscript sentence as recommended by reviewer.

**R#2-Comment-13-1: Fig S3E**. If these images were taken using confluent cell cultures, most of the cells are not GFP+ even after 40 days of hygromycin B selection. If this the case, it would question the meaning of every experiment that were performed with these cell lines.

R#2-Response-13-1: Once we established HEK293-EBV cells after 40 days of hygromycin B selection, we made cell stocks using nitrogen liquid. For experiments, we thawed the cell stock, picked up one single cell with the greatest GFP from the thawed cells, expanded the single cells in mass amount, and finally subjected most GFP+ cells in following experiments.

**R#2-Comment-14-1: Fig 6A**. Were the cells induced to measure extracellular EBV DNA?

R#2-Response-14-1: We analyzed EBV intracellular copy numbers and extra cellular copy numbers in both conditions such as no-induction and induction. Measurement of EBV copy numbers in no induced condition were posted in new Figure 6A. Furthermore, we measured EBV intracellular copy numbers and extra cellular copy numbers after treating 20 ng/ml TPA and 3 mM NaB for 48 hours. This induced data were also posted in new Figure 6A.

**R#2-Comment-14-2:** If not, how can EBV DNA be detected in supernatant?

R#2-Response-14-2: We do not know why EBV DNA could be detected but could detect very small amounts of EBV extracellular copy numbers when there was no induction. Whenever HEK293-EBV cells were died, EBV might be initiate its lytic cycles to produce EBV particles. The natural EBV particles might be detected in EBV copy number assay. For reference, we detected great amounts of EBV extracellular copy numbers with TPA/NaB induction.

**R#2-Comment-14-3:** Or B(+) EBV-transfected cells are lytic?

R#2-Response-14-3: We assumed in old manuscript that BART(+)·S13^-^ HEK293-EBV cells is more lytic than BART(+)·S13^-^ HEK293-EBV cells in the condition of no induction. To test this assumption, we compared their extracellular and intracellular EBV genome copy numbers in the condition of induction.

When cells were induced with TPA and NaB, extracellular EBV genome copies were not significantly different between BART(+)·S13^+^ HEK293-EBV cells and mutant BART(+)·S13^-^ HEK293-EBV cells, but extracellular EBV genome copies were huge different in BART(-)·S13^+^ HEK293-EBV cells and mutant BART(-)·S13^-^ HEK293-EBV cells (New Figure 6A).

However, when cells were not induced, extracellular EBV genome copies were significantly different between BART(+)·S13^+^ HEK293-EBV cells and mutant BART(+)·S13^-^ HEK293-EBV cells. Especially, mutant BART(+)·S13^-^ HEK293-EBV cells produced great amounts of extracellular EBV genome copies, relative to BART(+)·S13^+^ HEK293-EBV cells.

Similarly, mutant BART(+/-)·S13^-^ HEK293-EBV cells could enhance transcription of *BZLF1* lytic gene in induced condition with NaB and TPA (New Figure 6F), compared to wild-type BART(+/-)·S13^+^ HEK293-EBV cells. However, the BZLF1 upregulation was not great in mutant BART(+/-)·S13^-^ HEK293-EBV cells in non-induced condition (New Figure 6E).

Moreover, BART(+)·S13^-^ HEK293-EBV cells could not increase BZLF1 production in non-induced condition (New Figure 6G), compared to BART(+)·S13^+^ HEK293-EBV cells.

Based on these results, we speculated that BART(+)·S13^-^ EBV may be nonclassically biased to lytic replication but fail to complete lytic life cycle to make a functional EBV particles. In addition, we preferred the lytic induction as “spontaneous/abortive lytic reactivation” in mutant BART(+/-)·S13^-^ EBV.

**R#2-Comment-14-4: Fig 6B**. How is it determined based EBV BLLF1 gene southern blot which EBV genome is integrated into the human genome and which not?

R#2-Response-14-4: We picked up the *BLLF1* lytic gene as a probe to find EBV genomes in Southern blot assay. We also used BamH1 restriction enzyme and cleaved EBV genomes less than 11,867 bp as shown on below virtual gel. The BLLF1 gene locates at EBV genome 78803-79522 and on 5,053-bp fragment of below virtual gel. In new Figure 6B, Only EBV episome could produce Southern blot signal for 5,053-bp fragment. However, EBV genome is known to frequently integrate into at specific sites of human genome and modified by epigenetic effects. Thus, it is highly possible that top two signals larger than 8576 bp might be produced from EBV genomes integrated into human genome. At this moment, we could not explain where top two bands came from except EBV genome integrated to human genome.

**R#2-Comment-14-5: Line 296.** “The S13 locus is required for normal…” sentence needs to be revised using the name of the right EBV clones.

R#2-Response-14-5: We changed the sentence as recommended; “The S13 locus is required for normal expression of BART miRNAs in BART(-)·S13^+^ HEK293-EBV cells but efficiently suppresses BART miRNAs in BART(+)·S13^+^ HEK293-EBV cells.”

**R#2-Comment-14-6: Fig 6D**. Were these cells lytically induced or spontaneous/abortive lytic reactivation was measured?

R#2-Response-14-6: We measured spontaneous/abortive lytic reactivation in BART(+/-)·S13 HEK293-EBV cells and posted these results in new Figure 6. Mutant BART(+/-)·S13- HEK293-EBV cells were determined to cause spontaneous/abortive lytic reactivation in following experiments.

**R#2-Comment-15-1:** How the differential expression of genes in S13 mutant virus are connected to EBV 3D structure and looping between S13 locus and other parts of the EBV genome?

R#2-Response-15-1: We referred the EBV paper (“Hong-Shen Chen et al. J.Viol. 88(3): 1703-1713”). This paper reported that EBVΔCTCF166 caused to decrease total viable cells and elevate EBV episome copy numbers (Left lower figure). In addition, EBVΔCTCF166 showed severe reduction in DNA-DNA interaction between EBV genome 7-kb region and 166-kb region (Right lower figure). These phenotype of EBVΔCTCF166 was similar to the mutant BART(+)·S13^-^ EBV. Based on this similarity, we connected differential expression of genes in S13 mutant virus are connected to EBV 3D structure. However, we need to further study accurate mechanism about the involvement of S13 in regulating EBV gene expression and forming EBV 3D structures.

**R#2-Comment-16-1: Line 304-306.** Conclusion of experiments in **Fig 6** needs to be revised. The mutation of S13 site was used to determine what the role of S13 in wild type EBV. Thus, the results show the role of S13 locus in the regulation of BART miRNA expression and inhibition of lytic cycle and not the function of S13 mutation.

R#2-Response-16-1: We revised as reviewer’s suggestion; “These results indicate that the S13 locus plays a regulatory role in establishing EBV latency within the episome, regulating BART miRNA expression, and inducing EBV lytic reactivation”.

**R#2-Comment-17-1: Line 313-314.** “Compared with the mutant…” sentence needs to be revised using the name of the correct EBV clones. The same EBV clones are compared to the same virus clones?

R#2-Response-17-1: We revised as reviewer’s suggestion; “Compared with the wild-type BART(+/-)·S13^+^ HEK293-EBV cells, we found interesting results for mutant BART(+/-)·S13^-^ HEK293-EBV cells”.

**R#2-Comment-18-1: Line 324-325.** The conclusion sentence needs to be revised to make it more specific and meaningful.

R#2-Response-18-1: We revised reviewer’s suggestion; “Collectively, these epigenetic data favorably supports that CTCF bound on S13 locus plays dual roles in regulating EBV BART miRNA expression by adjusting adjacent CTCF distribution on S15 and S16 genomic loci.”

**R#2-Comment-19-1: Fig 6 data**, which showed increased lytic gene expression with S13 mutation in wt EBV, is in line with increased enrichment of RNAPII at S13 in B(+) Mt in Fig 7. Therefore, Fig 6 and 7 together indicate that CTCF-binding in the S13 locus favors less RNAPII binding, which correlates with less viral gene transcription. This is not the case when we compare B(-)wt with B(-)mut indicating the 11.8kb EcoRI fragment may affect the function of S13 locus in viral gene regulation.

R#2-Response-19-1: It is the most difficult question for us to answer this comment. Frankly speaking, we also do not understand why mutant BART(-)·S13^-^ EBV is not like mutant BART(+)·S13^-^ EBV. When we induced lytic reactivation by TPA/NaB, mutant BART(-)·S13^-^ EBV was severely defective in producing extracellular EBV genome copies but actively productive in producing intracellular EBV genome copies compared to wild-type BART(-)·S13^+^ EBV (new Figure 6A). However, mutant BART(+)·S13^-^ EBV was almost equally productive in producing extracellular EBV genome copies but severely defective in producing intracellular EBV genome copies compared to wild-type BART(+)·S13^+^ EBV (new Figure 6A). In addition, the role of S13 locus plays a positive transcriptional regulator for BART miRNA expression in wild-type BART(-)·S13^+^ EBV but a negative transcriptional regulator for BART miRNA expression in wild-type BART(+)·S13^+^ EBV. This difference of S13 in miRNA expression is attributed to the presence or absence of 11.8-kb EcoRI C fragment in EBV genome. We definitely assumed that 11.8-kb EcoRI C fragment has multifunctional roles in EBV life cycle by regulating EBV gene expression and forming EBV 3D genomic structure. It is required to further study about 11.8-kb EcoRI C fragment.

**R#2-Comment-20-1: General comment**: it is unclear how many times the ChIPs were done, how many biological replicates (n=?) were used to make the graphs. There are no significance tests anywhere in the paper. It should be added where it is appropriate.

R#2-Response-20-1: We did two independent ChIP experiments. Each ChIP experiment has three replicates of qPCR assay at test-points. We pooled two independent experiment sets whose results have three repetitions of the qPCR per a single ChIP experiment (thus, we obtained six results and processed them statistically). In addition, we added results of statistical significance test anywhere necessary in manuscript. Finally, we posted these modifications in new Figure 3 and 7.

**R#2-Comment-21-1: Fig 8.** In panel A, the 6-well pictures can be removed because they do not add anything to the figure.

R#2-Response-21-1: We transferred the old Figure 8A to the new supplemental Figure 4.

**R#2-Comment-21-2:** More accurate quantification is required than counting GFP positive cells in three different sections of the cell cultures.

R#2-Response-21-2: We presented three different sections of the cell cultures in the old Figure 8B, although their pictures were taken at different timepoints. One section taken at 24h p.i. is different from other two sections taken at 48h p.i. and 96h. p.i. We calculated GFP spots on these three sections and processed them statistically. To clarify how mutant EBV infects HEK293 cells, we normalized GFP spots with EBV virion produced from HEK293-EBV cells and made a new graph in new Figure 8C. Mutant BART(-)·S13- HEK293-EBV cells produced mutant EBV in very low amounts, and the mutant BART(-)·S13- EBV was a little defective to infect HEK293 cells compared to wild-type BART(-)·S13- EBV.

**R#2-Comment-21-3:** I recommend that they use qPCR for measuring viral DNA load in infected cells.

R#2-Response-21-3: We measured intracellular EBV genome copy numbers in infected BART(+/-)·S13 HEK293-EBV cells. Data of intracellular EBV genome copy numbers were posted in new Figure 8B, 8C.

**R#2-Comment-21-4:** Also, the question is whether virus production was reduced or the infectivity of the virus produced from B(+)Mut was decreased.

R#2-Response-21-4: We think that the infectivity of mutant BART(+/-)·S13^-^ EBV was highly decreased.

**R#2-Comment-21-5:** This result seems to contradict their previous results showing that S13 mutation in B(+) virus (SNU719) increases lytic gene expression.

R#2-Response-21-5: mutant BART(+)·S13- EBV is similar to EBVΔCTCF166 (“Hong-Shen Chen et al. J.Viol. 88(3): 1703-1713”). Both mutant EBVs increased their copy numbers but defective to survive in the infected cells, compared to wild-type EBV. We speculate that the upregulation of lytic gene expression in mutant BART(+)·S13- EBV might not produce normally infectious EBV particles but somehow defective EBV particles. However, we need to further study to clarify this argument.

**R#2-Comment-22-1: Fig 5D and 9A-B.** I recommend that they quantify their 3C-PCRs using qPCR including multiple biological replicates instead of uisng DNA agarose gels, which often show many different bands questioning specificity of the PCRs.

R#2-Response-21-4: We sequenced target DNA fragments indicating DNA interactions such as “3-kb ~ 135-kb” and “167-kb ~ 135-kb”. These sequencing data showed DNA regions linked between 135-kb region and two target regions such as 3-kb and 167-kb regions (Supplemental Figure 7). In addition, we added 4C-seq data showing DNA-DNA interactions in BART(+/-)·S13 HEK293-EBV cells in new Figure 9. However, we are reluctant to do qPCR assay due to two reasons. One is that there were multiple DNA bands on each 3C-PCR assay whose bands could attribute to false positive results on qPCR assay. The other is difficulty to design qPCR primer set unique to quantify only targeted DNA-DNA interactions. 3C products were concentrated DNA solution but target linked DNA fragments were very rare. Thus, it is required sometime to do nested PCR assay to pick up signals for DNA-DNA interactions. We did not use nested PCR assay but only exploited primary PCR assay to this analysis. qPCR assay might produce false positive results when it is applied to high concentration DNA solution. For this reason, we preferred check on gel the DNA product quality of 3C assay in each PCR experiment. This checking on gel let us be comfortable to use 3C DNA products in PCR assay for determining DNA-DNA interactions.

**R#3-Comment-1-1:** Reviewer #3: The authors should carefully delineate how many biological replicates were included in each experiment, and note, where appropriate, the statistical significance as determined by the methods stated in their methods section.

R#3-Response-1-1: We agreed to note statistical significance where necessary in manuscript (Fig4D,Fig6A,C,D,E,F, and Sup Fig4G), In addition, we added the number of biological replicates in figure legends (Fig1A, Fig3, 4, 6, 7, and 8)

**R#3-Comment-1-2:** Without these informations, it is very difficult to assess the rigor, reproducibility and validity of the data presented.

R#3-Response-1-2: We did two independent ChIP experiments. Each ChIP experiment has three replicates of qPCR assay at test-points. We pool two independent experiment sets whose results have three repetitions of the qPCR per a single ChIP experiment (thus, we obtained six results and processed them statistically). We added results of statistical significance test anywhere necessary in manuscript.

**R#3-Comment-1-3:** Are the western blots representative from 3 independent experiments?

R#3-Response-1-3: We repeated the Western blot five times and presented the best result in the old figure 6E. Most experiments were not successful to reach good results enough to go publication. Detection of EBV proteins in HEK293-EBV cells were difficult due to low expression levels of EBV proteins in the HEK293-EBV cells. We successfully detected EBV proteins three times, and representative images were shown in figures.

**Part III – Minor Issues: Editorial and Data Presentation Modifications**
Please use this section for editorial suggestions as well as relatively minor modifications of existing data that would enhance clarity.

**R#1-Minor comment-1-1:** Reviewer #1: The manuscript needs extensive editing to remove ambiguities and English language errors.

R#1-Response to minor comment-1-1: We asked English editing agent to intensively revise our manuscripts and sincerely revised as reviwer’s recommendations to remove ambiguities and English language errors.

Reviewer #2: (No Response)

**R#3-Minor comment-1-1:** Reviewer #3: The manuscript is well written, but there are areas of text in the results that are pretty sparse in detail. This makes it difficult for a broad audience to grasp the depth of the experimental findings. For example,

Figure 2- This series of experiments, and what the output data means is confusing. Perhaps it is my unfamiliarity with the specifics of the experiment, but the authors should consider adding text to this section to carefully explain what the MST assay is, what it detects and how this is relevant to the results presented.

R#3-Response to minor comment-1-1: We added text to carefully explain what the MST assay is, what it detects and how this is relevant to the results presented in manuscript.

other suggestions:

**R#3-Minor comment-2-1:** Figure 5. 4C-plots. These are not easy to interpret, especially if the reader is unfamiliar with the EBV genome. The long-range interactions shown are fine, but it would be helpful if a) the EBV genome map was legible on the outside of the Circos plot and b) if the authors helped to identify areas that they specifically discuss in the text (with an arrow maybe on the figure?).

R#3-Response to minor comment-2-1: We added key features on EBV genome on the outside of the Circos plot and marked areas that we specifically discuss in the text.

**R#3-Minor comment-3-1:** Figure 1: how many reps were done for ChIP seq?

R#3-Response to minor comment-3-1: We did B95-8 ChIP-seq two times and SNU719 ChIP-seq three times. Now, the number of biological replicates were added in figure legends (Fig1A, Fig3, 4, 6, 7, and 8).

**R#3-Minor comment-4-1:** Figure 3: Stats? Also, how were the ChIP assays validated? IgG? another set of cellular controls?

R#3-Response to minor comment-4-1: We always added IgG negative control in all the ChIP assays.

**R#3-Minor comment-5-1:** Fig 4: Stats? Replicates?

R#3-Response to minor comment-5-1: We did two independent miRNA RT-qPCR experiments. Each miRNA RT-qPCR experiment has three replicates of qPCR assay at testing miRNAs. We pool two independent experiment sets whose results have three repetitions of the qPCR per a single miRNA RT-qPCR experiment (thus, we obtained six results and processed them statistically). We added results of statistical significance test anywhere necessary in manuscript.

**R#3-Minor comment-6-1:** Fig 7- same comments as figure 3 (see above)

R#3-Response-1-2: We did two independent ChIP experiments. Each ChIP experiment has three replicates of qPCR assay at test-points. Regarding old Figure 7. We presented the best result well fitted to other experiment data between the two ChIP experiments. However, we modified Figure 7 as recommended by reviewer #1. We pool two independent experiment sets whose results have three repetitions of the qPCR per a single ChIP experiment (thus, we obtained six results and processed them statistically). We added results of statistical significance test anywhere necessary in manuscript.

PLOS authors have the option to publish the peer review history of their article ([what does this mean?](https://journals.plos.org/plospathogens/s/editorial-and-peer-review-process#loc-peer-review-history)). If published, this will include your full peer review and any attached files.


If you choose “no”, your identity will remain anonymous but your review may still be made public.


**Do you want your identity to be public for this peer review?** For information about this choice, including consent withdrawal, please see our [Privacy Policy](https://www.plos.org/privacy-policy).

Reviewer #1: No

Reviewer #2: No

Reviewer #3: No

Figure Files:

While revising your submission, please upload your figure files to the Preflight Analysis and Conversion Engine (PACE) digital diagnostic tool, [https://pacev2.apexcovantage.com](https://pacev2.apexcovantage.com/). PACE helps ensure that figures meet PLOS requirements. To use PACE, you must first register as a user. Then, login and navigate to the UPLOAD tab, where you will find detailed instructions on how to use the tool. If you encounter any issues or have any questions when using PACE, please email us at [figures@plos.org](mailto:figures@plos.org).

Data Requirements:

Please note that, as a condition of publication, PLOS' data policy requires that you make available all data used to draw the conclusions outlined in your manuscript. Data must be deposited in an appropriate repository, included within the body of the manuscript, or uploaded as supporting information. This includes all numerical values that were used to generate graphs, histograms etc.. For an example see here on PLOS Biology: http://www.plosbiology.org/article/info%3Adoi%2F10.1371%2Fjournal.pbio.1001908#s5.

Reproducibility:

To enhance the reproducibility of your results, we recommend that you deposit your laboratory protocols in protocols.io, where a protocol can be assigned its own identifier (DOI) such that it can be cited independently in the future. Additionally, PLOS ONE offers an option to publish peer-reviewed clinical study protocols. Read more information on sharing protocols at <https://plos.org/protocols?utm_medium=editorial-email&utm_source=authorletters&utm_campaign=protocols>

*In compliance with data protection regulations, you may request that we remove your personal registration details at any time.* [*(Remove my information/details)*](https://www.editorialmanager.com/ppathogens/login.asp?a=r)*. Please contact the publication office if you have any questions.*
