# Supplementary material for: Characterization of a new CCCTC-binding factor binding site as a dual regulator of Epstein-Barr virus latent infection
Source: PLoS Pathog. 2023 Jan 25;19(1):e1011078. doi: 10.1371/journal.ppat.1011078 (PMC9876287; doi:10.1371/journal.ppat.1011078)
Supplement: Attachment — Submitted filename: Responses to Reviwers comments_2nd revision.docx [file ppat.1011078.s019.docx]

Responses to reviewer’s’ comments

We answered line by line to reviewer’s all concerns and sincerely revised as they suggested for the publication of our manuscript to PLOS PATHOGENS. Beside to conducting experiments reviewers requested, we repeated CTCF ChIP-seq assays using B95-8 and SNU719 cells and revised Figure 1A with these new data. At last, we revised Fig.10 to make it look visually better.

The revised manuscript is much improved, but there remain critical concerns, both in terms of writing as well as specific experiments, that affect rigor and reproducibility.

Each experiment must be rigorous even if it is meant to only support other experiments.

Please more thoroughly address the remaining reviewer concerns.

All of those comments should be addressed, but I would editorially advise in particular, though not exclusive attention to:

1) removal of any experiments without adequate controls and any experiments that are not convincing (the Southern blot appears both not reproducible and perhaps incorrectly interpreted; the 3C assays are "variable" and have no negative control)

1. We repeated Southern blot assay and obtained results similar to previous experiments. However, we also agreed to incorrectly interpret the Southern blot results and removed all comments about genomic integration of EBV episomes. We only addressed the Southern blot result to confirm the establishment of EBV episomes in HEK293-EBV cells. In addition, we moved the Southern blot result to supplemental Figure 5F.
2. We performed negative experiments for 3C-PCR assays and posted these results in supplemental figure 3 and 10.

2) more clear explanation of how sequencing data confirms deletion of the EcoRI C fragment in B95-8 cells

1. We did not sequence whole genomes of B95-8 EBV and GC1(SNU719) EBV but acquired their genome sequences from GeneBank for pairwise alignment analysis.
2. Several scientists such as Drs. Griffin, Barrell, and Hayward reported that B95-8 EBV genome has a deletion of the EcoRI C fragment in their genome. In particular, the B95-8 EBV genome is reported as GenBank number V01555.2 in which the deleted EcoRI C fragment could be marked by comparing the EBV Wt genome tagged as GenBank number NC_007605.
3. We observed the deletion in B95-8 EBV genome (GeneBank number V01555.2) comparing GC1 (=SNU719) EBV genome (GeneBank number KP735248) by pairwise alignment of EBV genomes (reference: Journal of microbiology, 2018, 56(8): 525-533).

(Journal of microbiology, 2018, 56(8): 525-533)

1. Experimentally, we observed no expression of BART6-5p and BART11-5p miRNAs in B95-8 EBV genome but clear expression of the BART6-5p and BART11-5p miRNAs in GC1(SNU719) EBV genome (refer Fig 4B and 4C).
2. Based on reference data and experimental results, we are confident that there is a deletion of the EcoRI C fragment in B95-8 cells.

3) more clear organization of the MST data including addition of a Coomassie gel of purified CTCF (not just the overexpressed Sf9 lysate).

1. We replicated the Coomassie gel of Sf9-CTCF lysate with the new Coomassie gel of purified CTCF in Fig 2A.
2. We also calculated Kd value in MST control experiment with Wt S13 oligo and Sf9 lysate. The Kd value was 6.0 ± 3.5 µM.

There is a lot of data presented in the revision, but for the next version please emphasize quality over quantity to facilitate a final decision from the journal.

Reviewer #1: Comment 1: The authors make several comparisons between B95-8 and SNU719 that are directly attributed to the deletion reported to be in B95-8 e.g. Lines 184-186: “Considering that the 11.8-kb EcoRI C fragment (EBV 139724~151554) exists only in the SNU719 EBV genome, it may play an unfavorable role in recruiting CTCF and RNAP II in the SNU719 EBV genome”. How can these comparisons be substantiated if the EcoRI C fragment deletion appears to be in both cell lines? If they cannot resolve the sequencing data to show that the EcoRI C fragment exists in SNU719 and not B95-8, then such comparisons cannot be drawn.

1. We did not sequence whole genomes of B95-8 EBV and GC1(SNU719) EBV but acquired their genome sequences from GeneBank for pairwise alignment analysis.
2. Several scientists such as Drs. Griffin, Barrell, and Hayward reported that B95-8 EBV genome has a deletion of the EcoRI C fragment in their genome. In particular, the B95-8 EBV genome is reported as GenBank number V01555.2 in which the deleted EcoRI C fragment could be marked by comparing the EBV Wt genome tagged as GenBank number NC_007605.
3. We observed the deletion in B95-8 EBV genome (GeneBank number V01555.2) comparing GC1 (=SNU719) EBV genome (GeneBank number KP735248) by pairwise alignment of EBV genomes (reference: Journal of microbiology, 2018, 56(8): 525-533).

(Journal of microbiology, 2018, 56(8): 525-533)

1. Experimentally, we observed no expression of BART6-5p and BART11-5p miRNAs in B95-8 EBV genome but clear expression of the BART6-5p and BART11-5p miRNAs in GC1(SNU719) EBV genome (refer Fig 4B and 4C).
2. Based on reference data and experimental results, we are confident that there is a deletion of the EcoRI C fragment in B95-8 cells.

Fig 2A and S6b: A molecular weight marker is still not indicated on the western blots shown in Fig 2A. Line 149 “CTCF protein was of good quality and suitable for subsequent experiments.” By what criteria? The Coomassie stained gel now included (fig S6b) shows the protein is highly contaminated. In fact, compared to the Sf9 lysate there really is no further purification of CTCF protein. The use of such impure protein preparations in sensitive in vitro assays such as MST can give spurious and misleading results. This is demonstrated by the small difference in calculated Kd between wtS13 + CTCF and mtS13 + Sf9, which presumably is not significant judging by the reported standard deviations. Kd values should be reported for all four experimental conditions.

1. We replicated the Coomassie gel of Sf9-CTCF lysate with the new Coomassie gel of purified CTCF in Fig 2A.
2. We also calculated Kd value in MST control experiment with Wt S13 oligo and Sf9 lysate. The Kd value was 6.0 ± 3.5 µM.

Line 294: still references Fig S4B – should be Fig S4G

1. We fixed this error as recommended.

Comment 5-1 – has not been fully addressed – “The 3C experiments shown in Fig5D and Figure 9 would benefit from a figure explaining the method and location of primers used.”

1. We could not make other markers in 4C-seq plots to point locations of 4C-seq baits because the plots have already several notes in quite narrow spaces.
2. Instead of adding new notes in 4C-seq plots, we addressed locations of 4C-seq baits in legends of figures such as Figure 5, Figure 9, supplemental Figure 1B, and supplemental Figure 8.
3. In supplemental Figure 1B and 8, we wrote “The FR viewpoint primer sets were located at EBV 4731–4751 and EBV 4941–4961” to address the location of 4C-seq FR bait.
4. In Figure 5 and 9, we wrote “Locations of viewpoint primer sets were posted in S3 Table; S13 (EBV 138504 ~ 138987), BART-1 (EBV 147357 ~ 147490), LMP1/2 (EBV 166480 ~ 166661), Qp (EBV 50278 ~ 51579), OriP (EBV 9918 ~ 10302) and FR (EBV 4731 ~ 4961)” to address the location of 4C-seq baits.

Comment-5-2: There do not appear to be any positive or negative controls for this experiment (now fig 5F and G)

1. We used as positive control the DNA interaction between 3-kb and 167-kb regions in supplemental Figure 2A, supplemental Figure 2B, and supplemental Figure 9B.
2. We again conducted all sets of negative control experiments with unligated Xho1-digested DNA for 3C-PCR assays and posted these negative control results in Supplemental Figure 3 and Supplemental Figure 10.

Response-5-2: We used “DNA link between 3-kb and 167-kb” as “positive control” in this 3C-PCR 202 assay. “DNA link between 3-kb and 167-kb” was reported in previous studies (reference: attached 203 below). But we did not include any special negative control. 3C-PCR assay showed variable data in our hands. Actually, we observed slightly different results in each 3C-PCR assay and not such convincing these 3C-PCR data.
This comment has not been adequately addressed – the experiment needs a robust negative control. The fact that the authors have declared the variability in their experiment makes me doubt the results further.

1. We used as positive control for the DNA interaction between 3-kb and 167-kb regions in supplemental Figure 2A, supplemental Figure 2B, and supplemental Figure 9B.
2. We again conducted negative control experiments for 3C-PCR assays and posted these negative control results in Supplemental Figure 3 and Supplemental Figure 10.

Comment 6-2: Now referring to lines 341-344. The densitometry shown is really difficult to interpret. The analysis is outside of the linear range of detection (indicated by maximum intensity being reached in almost all of the bands in the second repeat).

1. This table is the best densitometry DATA in which we can calculate band intensity using ImageJ software.
2. Small number in band intensity table is indicating weak/white band on Southern blot plot and big number in band intensity table is indicating thick/dark band on Southern blot plot.
3. Please utilize these numbers in S7 table to recognize band intensity on Southern blot plot and relatively compare intensity among bands.

It also appears that the authors have cherry picked the blots they analysed – 3/5 blots were excluded because they obtained the ‘best result’ from southern blots 4 and 5. They should either remove these data or provide better analysis.

1. We repeated Southern blot assay and obtained results similar to previous experiments. However, we also agreed to incorrectly interpret the Southern blot results and removed all comments about genomic integration of EBV episomes. We only addressed the Southern blot result to confirm the establishment of EBV episomes in HEK293-EBV cells. In addition, we moved the Southern blot result to supplemental Figure 5F.
2. We performed negative experiments for 3C-PCR assays and posted these results in supplemental Figure 3 and 10.

Comment 7: so this is an n=2 experiment. Statistical analysis should be performed as such otherwise you are including the error in the PCR step, not the ChIP experiment per se.

1. We repeated (n=2) whole experiments to make meaningful statistics; from cell culture, through ChIP experiment, to qPCR analysis.
